# Identification of critical amino acids in the DNA binding domain of LuxO: Lessons from a constitutive active LuxO

**DOI:** 10.1371/journal.pone.0310444

**Published:** 2024-09-17

**Authors:** Shradha Surin, Richa Singh, Manpreet Kaur, Gourab Basu Choudhury, Himanshu Sen, Chetna Dureja, Saumen Datta, Saumya Raychaudhuri

**Affiliations:** 1 CSIR-Institute of Microbial Technology, Sector 39A, Chandigarh, India; 2 Academy of Scientific and Innovative Research (AcSIR), Ghaziabad, India; 3 CSIR-Indian Institute of Chemical Biology, Jadavpur, Kolkata, India; Gandhi Insititute of Technology and Management, INDIA

## Abstract

Quorum sensing plays a vital role in the environmental and host life cycles of *Vibrio cholerae*. The quorum-sensing circuit involves the consorted action of autoinducers, small RNAs, and regulatory proteins to control a plethora of physiological events in this bacterium. Among the regulatory proteins, LuxO is considered a low-cell-density master regulator. It is a homolog of NtrC, a two-component response regulator. NtrC belongs to an evolving protein family that works with the alternative sigma factor σ^54^ to trigger gene transcription. Structurally, these proteins comprise 3 domains: a receiver domain, a central AAA+ATPase domain, and a C-terminal DNA-binding domain (DBD). LuxO communicates with its cognate promoters by employing its DNA binding domain. In the present study, we desired to identify the critical residues in the DBD of LuxO. Our combined mutagenesis and biochemical assays resulted in the identification of eleven residues that contribute significantly to LuxO regulatory function.

## Introduction

Cholera is a severe diarrheal disease of global importance. Even in the 21^st^ century, some parts of the globe continue to face the scourge of cholera [1,2]. *Vibrio cholerae* is the causative agent of this deadly disease. The organism possesses a wide array of virulence factors and has a tremendous ability to sustain itself in diverse aquatic environments [3]. Both the survival and pathogenesis of the organism are superbly coordinated by a cellular mechanism known as quorum sensing [4]. Decades of research on the quorum-sensing sensory network of *V*. *cholerae* have garnered a large swath of data on the interplay and functionality among myriad factors, including small metabolites, non-coding RNAs, and regulatory proteins of the sensory circuit. Among various regulatory proteins, LuxO is known as a low cell-density master regulatory protein. At low cell density, LuxO is activated through phosphorylation, and activated LuxO~P in collaboration with sigma-54 and Fis stimulates the production of a cascade of small RNAs designated as Qrr (quorum response regulatory) RNA. The Qrrs affect the stability of mRNA of HapR, a high cell density master regulator and enhance expression of AphA LuxO, the low cell density master regulator, activates the production of primary virulence factors, promotes biofilm development, and affects protease production through AphA and HapR. By modulating HapR function, LuxO controls the physiology and pathogenesis of the organism to a larger extent at low cell density. The situation inverts at high cell density when LuxO gets dephosphorylated and dephosphorylated LuxO is incapable of suppressing HapR function [5]. HapR, the high cell density master regulator, directly stimulates protease production by interacting with the cognate promoter of the gene (*hap*A) encoding the HapA protease. HapR also affects virulence and biofilm development at high cell density. In short, the life cycle of *Vibrio cholerae* largely depends on the temporal balance between these two regulatory proteins in a density-dependent manner.

**Fig 1:**
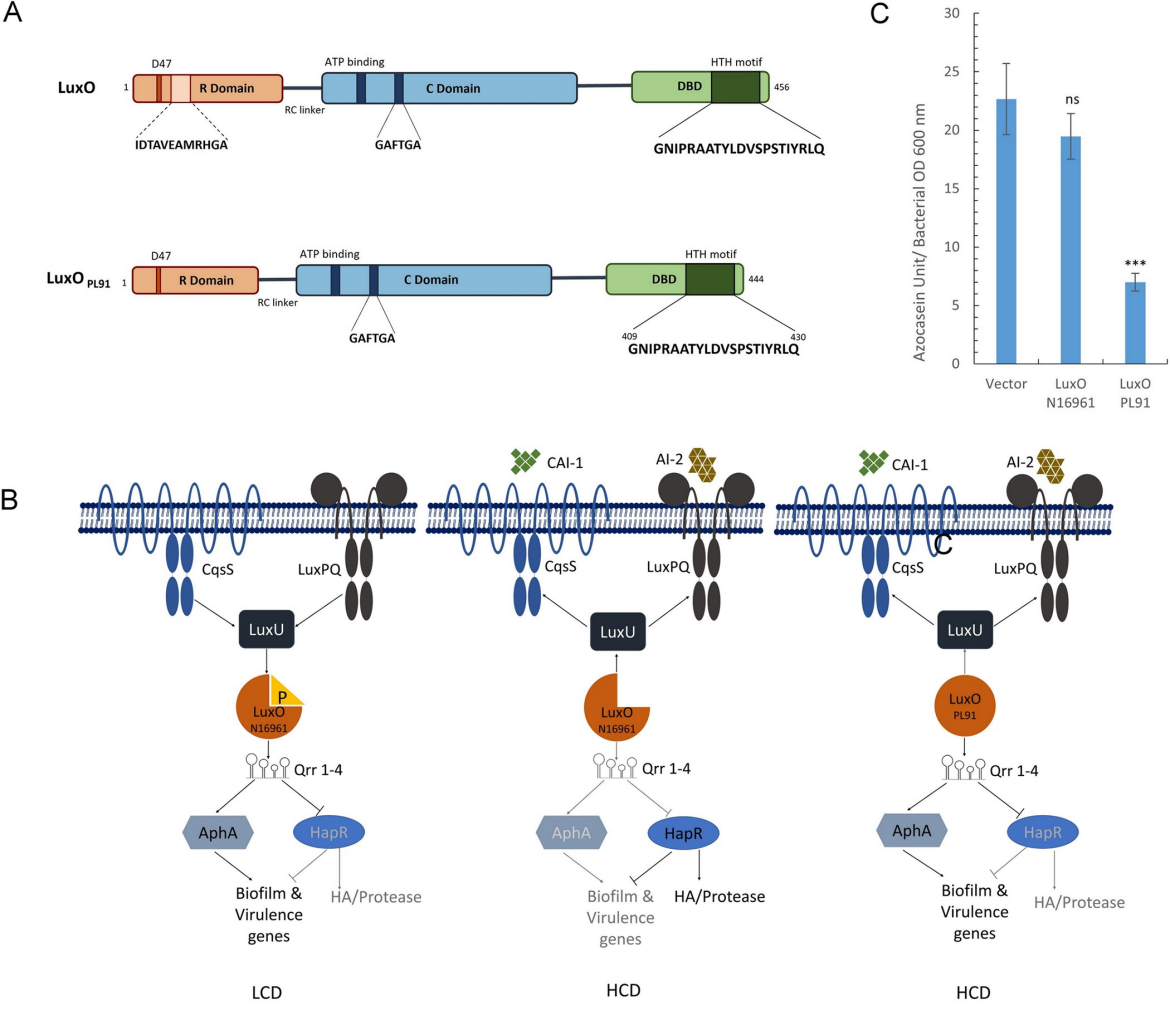
Domain structure and constitutive behaviour of LuxO_PL91_. (A) LuxO comprises of three domains: the N-term Receiver (R) domain, the central AAA+ ATPase (C) domain and C-term DNA binding Domain (DBD). LuxO_PL91_ consists of a 12 amino acid deletion in the R domain and shows a constitutive behaviour (B) Quorum Sensing pathways followed at Low Cell Density (LCD) phase and High Cell Density (HCD) by *Vibrio cholerae*. The constitutively active LuxO (LuxO_PL91_) bypasses the need for phosphorylation and thus even at HCD stays active and follows the LCD circuit (C) Cell free supernatants from overnight cultures of MM307 carrying pKK-3RI (Vector), LuxO_N16961_ and LuxO_PL91_ grown in TSB-D were checked for the proteolytic activity via digestion of azocasein. The enzymatic activity was calculated from the average of two independent experiments (n = 6) and is plotted as mean ± SD. One way ANOVA was used for statistical analysis wherein (*—P < 0.05; **—P < 0.01; ***—P < 0.001; ns—not significant).

LuxO is a member of the subfamily of AAA+ ATPases bacterial enhancer-binding proteins (bEBPs) [6–8]. The members of this family carry out diverse molecular functions, including transcriptional regulation at the expense of ATP hydrolysis [9,10]. Like members of the bEBP family who promote transcriptional regulation through DNA unwinding [11], LuxO also stimulates the “opening” of σ^54^-dependent promoters and further transforms such promoters into a transcriptionally active state [8]. Structurally, proteins (e.g., LuxO and NtrC) belonging to the bEBP family are segmented into three domains as N-terminal receiver (R) domain, a central ATPase (C) domain, and a C-terminal DNA binding (D) domain **(Fig 1A)** [6–8]. Mechanistically, the R domain inhibits the development of active oligomers by stabilizing the inactive dimers. Phosphorylation of the R domain relieves the inhibition, and subsequently, the C-domain drives the formation of active oligomers [12–15]. In case of LuxO, the transcriptional regulatory activity is also linked with the phosphorylation of an aspartate residue (D47) at the R domain of the LuxO. The phosphorylation further stimulates ATP hydrolysis through the C-domain, followed by the opening of σ^54^-dependent promoters and the production of copious amounts of Qrrs to control quorum sensing at low cell density [5,8]. In other words, phosphorylation plays a key role in nullifying the negative influence of the R domain over the C-domain, resulting in the subsequent activation of LuxO. Several studies have reported mutations in the R domain that converts LuxO into a constitutively active state that does not require phosphorylation and can function even at high cell density [16–18]. Bassler and group showed that substitutions in R domain residues Asp47 to Glu and Phe94 to Trp converts it into a constitutive state [16]. Further a natural variant of LuxO harbouring an L104Q is also shown to be a constitutively active [17]. In fact, the removal of the entire R domain transforms *Vibrio harveyi* LuxO into a constitutively active state [16]. To further gain insight into LuxO function, the crystal structure of *Vibrio angustum* LuxO was resolved in the recent past [8]. Though the structure does not include a DNA binding (D) domain, it has both R and C domains [8]. Interestingly, *V*. *angustum* LuxO monomer forms continuous helical arrays with six subunits per turn instead of forming closed rings as evidenced in some members of AAA+ ATPase family. The R domain is positioned between the two lobes of the C domain with distinct interfaces. Interaction between α-helix 5 (α5) of the R domain and α-helix 7 (α7) of the C domain occurs at interface I while interface II serves as a platform for the interaction among α4 and β5 of the R domain with α13 of the C domain [8]. A linker comprises of 20 amino acid residues designated as R-C linker (residues 123–142) connects both the R and C domains. Intriguingly, binding of the ATP substrate at the C-domain active site is occluded by a portion of the R-C linker (residues 137–142), which occupies the active site of the C-domain. The most important residue turns out to be a glycine moiety at position 141 (Gly 141) that stabilizes the interaction of RC linker residues with the active site of the C-domain. The unique positioning of the R-C linker into the active site is exclusively witnessed in LuxO but not with another member of the bEBP family proteins [8]. We learn from the previously reported σ^54^ associated transcriptional regulators that it binds with enhancer sequences upstream of the σ^54^ promoter [19,20]. Hence, it can be said that the transcriptional regulations through σ^54^ promoter region unwinding is mainly initiated by LuxO’s C-terminal DNA binding domain. The successful binding of the C terminal DBD helps proper placement of the R and C domain of the protein. These two domains (R and C) of the LuxO subsequently take part in activating σ^54^ polymerase to start transcriptions. Additionally, LuxO’s structural homologs like NtrC DBD has been reported to bend the DNA close to approximately 9° that ensures efficient phosphorylation and subsequent catalysis by RNA polymerase [21]. So, the DNA conformation at the enhancer region may also change before the unwinding as a result of successful DNA protein interaction.

Interestingly, natural variants of quorum sensing master regulators (e.g., LuxO and HapR) are quite frequent in *Vibrio cholerae* [17,18,22–25]. The existence of particular mutant forms of LuxO, otherwise known as constitutively active LuxO (con-LuxO), is reported in the literature. Such hyperactive LuxO variants remain active at high cell density and locks the cellular physiology at the low cell density phase **(Fig 1B)** [17,26]. Strains harbouring con-LuxO have been reported to develop very strong biofilm, (e.g., pellicle formation) [5,17,18] at high cell density. High biofilm development contributes to the better environmental survival of such strains. Such strains are also more virulent because of the upregulation of toxins and toxin-coregulated pili and exhibited protease negative phenotype. Deletion of such LuxO renders the strain to become protease positive at high cell density [17,18]. The transcriptome analysis of the *V*. *cholerae luxO* mutant strain exhibited a spectrum of differential gene expression, including genes encoding metabolic enzymes [4]. Therefore, it is conceivable that cellular energy status will also be modulated in the presence of constitutively active LuxO.

LuxO contains an HTH DNA binding domain at the C-terminal [20]. Previously, we identified a natural hyperactive variant of LuxO (henceforth designated as LuxO_PL91_) that harbours a deletion of twelve amino acids in the N-terminal receiver domain of the molecule **(Fig 1A)** [18]. In the present work, we employed a systemic alanine scanning mutagenesis on the C terminal DBD domain of LuxO_PL91_ to evaluate the contribution of each amino acid in LuxO regulatory function. Our mutagenesis, coupled with biochemical analysis of each alanine variant, aids in the identification of eleven amino acids contributing to LuxO DNA binding regulatory activity.

## Materials and methods

### Bacterial strains and growth conditions

**S1 Table** includes the list of bacterial strains and plasmids used in this study. All the strains were propagated at 37°C in Luria Bertani (LB) broth with agitation or on LB agar unless otherwise mentioned. *Escherichia coli* strain Nova Blue was used for cloning purposes. *Vibrio cholerae* recombinant strain MM307 (C6706 carrying in-frame LuxO deletion) was primarily used in this study. MM307 harbouring the LuxO DBD alanine mutants in pKK177-3RI vector background was used for the assays. For overexpression of proteins, the LuxO DBD alanine variants were cloned in an overexpression vector, pET28C, which was expressed in *E*. *coli* strain BL21(DE3). Ampicillin (0.1 mg/ml), streptomycin (0.1 mg/ml), and kanamycin (0.05 mg/ml) were added to the media accordingly. All antibiotics were procured from Sigma-Aldrich (MO, USA), culture media components were procured from BD Difco™ (NJ, USA), sheep blood agar plates (MP144), and Barritt reagents A and B were purchased from HiMedia (Mumbai, MH, India).

### Protease assay

Protease enzyme activity was determined using an azocasein digestion-based assay, as previously mentioned in [27]. Briefly, MM307 recombinant strains were grown into in tryptic soy broth without D-glucose (TSB-D), with appropriate antibiotics and shaking until the stationary phase reached 37°C. To 0.1 mL of stationary phase culture supernatant, 0.1 mL of azocasein (Sigma-Aldrich) (5 mg/mL in 100 mM Tris pH 8.0) was added and incubated at 37°C for 1 hr. The reaction was terminated by adding 0.4 mL of 10% trichloroacetic acid (Sigma-Aldrich). After centrifugation, the supernatant was collected and then transferred to 0.7 mL of 525 mM NaOH (Merck & Co.), and the absorbance at 442 nm was noted. One azo-casein unit is defined as the amount of protease producing an increase of 0.01 OD units/hr.

### 3D modelling of C-terminal LuxO and DNA binding analysis

We employed ColabFold2 (https://github.com/sokrypton/ColabFold) to make a 3D model of the C-terminal region of LuxO having 78 residues (366–443). Colabfold2 is based on AlphaFold2 and RoseTTAFold, accelerated by a fast multiple sequence alignment (MSA). We uploaded LuxO C-terminal sequences and performed multiple cycles with ColabFold2 on Google Colab to generate 5 different predicted PDB files assessed by pLDDT and pTMscore.

Next, with the Dali server [28] which searches for similar structures available in the Protein Data bank, we compared the Colabfold model of the protein. We also identified a few DNA-bound structures similar to those found in the same search. All the essential similar structures having maximum sequence identity is tabulated.

Structural analysis for DNA protein interaction was done in PyMOL by superimposing the model C-terminal LuxO with NtrC4, DeoR, and Fis DNA binding protein having PDB ID 4FTH, 7BHY, and 3IV5. Then, we created a DNA protein complex with our protein of interest, LuxO DBD. The complexes were employed for energy minimization in HADDOK (version 2.4) web server [29,30].

### Site-directed mutagenesis

All LuxO DBD alanine mutants were generated using Stratagene’s Quick Change PCR kit as published company protocol using the primers listed in the **S2 Table**. All clones carrying the desired mutations were confirmed by sequencing in their entirety. The desired clones were transformed in *V*. *cholerae* strain MM307.

### Colony morphology and biofilm formation

Colony morphology was studied with slight modifications in the published protocol [31]. Overnight grown cultures of *V*. *cholerae* recombinant strains were spotted on an LB agar plate containing appropriate antibiotics and incubated at 37°C for 24 hr. The appearance of rugose and smooth colonies was visualized photographically.

Biofilm formation assay was set up as described previously with minor changes [32]. Overnight grown cultures were diluted in a ratio of 1:100 and grown until OD_600nm_ 0.4–0.6. LB contained in borosilicate glass tubes was inoculated with 10^6^ cells and grown in static conditions for 18 hr at 37°C. The biofilm formed was washed twice with 1X phosphate-buffered saline (PBS) and stained with 0.1% crystal violet (HiMedia). An image of the stained biofilms was then taken using the Canon PowerShot SX510 HS camera.

### Haemolysin production and activity

Haemolysin production and activity were measured as previously described [33]. All *Vibrio cholerae* MM307 recombinant strains carrying LuxO_PL91_ DBD mutants were grown overnight in LB media. The overnight cultures were diluted 1:100 in fresh media and grown further till early log phase. Subsequently, 10^7^ cells were then dispensed into wells created in sheep blood agar plates (HiMedia). The zone of clearance was measured after 18 hours of incubation, and the image was taken using the Canon PowerShot SX510 HS camera.

### Voges-proskauer (VP) test

Voges-Proskauer (VP) test was carried out as explained earlier with some changes [34]. Briefly, all *Vibrio cholerae* strains were grown in LB media. A secondary culture was set up at 0. 01 OD_600nm_ in LB supplemented with 1% glucose (LBG) and allowed to grow for 12 hrs. This was followed by performing the Voges Proskauer (VP) test and spot-dilution of the harvested samples. For the VP test, 100 μl of culture was mixed with 20 μl of Barritt Reagent A (HiMedia) and 10 μl of Barritt Reagent B (HiMedia), and incubated at room temperature, following which image was taken.

### Western blot

Flag-tagged LuxO DBD variants were grown in TSB-D media, as mentioned previously [35]. Overnight cultures were harvested to get protein samples from an equal number of cells, which were then separated electrophoretically on 12% polyacrylamide sodium dodecyl sulphate gel. The proteins were then transferred to a PVDF membrane (GE Healthcare, Chicago). The membrane was then blocked using 5% skim milk in PBS. The membrane was washed using 1X PBST seven times each for 10 minutes, followed by incubation with an anti-FLAG Peroxidase (HRP) conjugated monoclonal antibody (Sigma). For loading control, the membrane was incubated with anti-RNA polymerase ß primary antibody (Abcam, Cambridge). The membrane was washed again and incubated with Goat anti-rabbit HRP conjugate IgG secondary antibody (GeNei^TM^). The protein bands were visualized using the Luminata Forte Western HRP substrate (Merck) on Azure 280 (Azure Biosystems, Inc. CA, USA).

### Protein purification & EMSA

*Vibrio cholerae* LuxO_PL91_ and its DBD alanine mutants, showing loss in activity were selected for protein overexpression. For protein purification, the gene encoding for LuxO_PL91_, LuxO_G409A_, LuxO_N410A_, LuxO_I411A_, LuxO_L418A_, LuxO_V420A_, LuxO_S423A_, LuxO_I425A_, LuxO_Y426A_, LuxO_R427A_, LuxO_K428A_ and LuxO_L429A_ were cloned in NdeI/XhoI sites of pET28c (S1 Table), thereby generating a N terminal 6XHis-LuxO fusion proteins. These were then transformed into BL21(DE3) cells for protein overexpression.

After induction with 0.2 mM Isopropyl ß-D-1-thiogalactopyranoside, all recombinant proteins were purified using Ni- NTA Agarose beads (Qiagen, Hilden, Germany). All proteins were dialyzed using a buffer containing 30 mM Tris-HCl pH 8.0, 300 mM KCl, 1 mM EDTA.

The protein oligomerization state was checked by electrophoresing the purified proteins on 12% polyacrylamide gel with or without dithiothreitol (DTT) at 40 mM as per the published protocol [35,36]. Gels were stained with 0.25% Coomassie brilliant blue R250 (Biorad) made in 45% methanol and 10% acetic acid. Destaining of the gels was also carried out in the same buffer without Coomassie brilliant blue.

Gel mobility shift assay was done as described [37]. The promoter region of Qrr4 (pQrr4) of length 523 bp was amplified with primer pairs as listed in **S2 Table**. The fragments were gel-purified and end-labelled with [γ 32P] dATP using T4 polynucleotide kinase (New England Biolabs, US). The binding reaction was carried out with 4 ng of labelled fragment in 10 mM Tris-HCl (pH 7.9), 0.1 mM EDTA, 1 mM DTT, 300 mM KCl, 1 mM MgSO4, 10% glycerol, 20 ug of BSA, and 0.5 ng of poly(dIdC) in a 20 μL reaction volume for 40 min at 26°C. The reaction mixture was applied to a 5% polyacrylamide gel (1X TAE pH 8) and subjected to electrophoresis in 1X TAE buffer, pH 8.5, at 4°C. The gel was dried using a gel dryer (Biorad Model 583) and autoradiographed to examine the shift of the band.

## Results

### Structural insight into the HTH domain of LuxO

Being a member of the NtrC activator family, LuxO directly activates the transcription of a group of small RNAs (Qrrs) by communicating with the promoter region. As described in the introduction, the Qrrs are expressed at low cell density and control the expression of HapR and HapR-regulated physiological phenomena. Therefore, functional modulation of LuxO mutants could be monitored by examining the cellular events governed by Qrrs either directly or indirectly through HapR. Notably, constitutively active LuxO (a hyperactive variant LuxO) maintains the production of Qrr small RNAs even at high cell density and reverts HapR-driven cellular physiology [5,17,18,26].

*Vibrio cholerae* strain PL91 carrying a hyperactive LuxO (LuxO_PL91_) is non-responsive to quorum sensing mediated cellular events and exhibits altered phenotypic traits by inhibiting HapR function at high cell density **(Fig 1B)** [18,38]. In the present work, we exploited this con-LuxO variant to create LuxO DBD alanine mutants. We hypothesized that those LuxO_PL91_ DBD mutants that fail to produce Qrrs at high cell density would also be defective in suppressing HapR-governed cellular properties at high cell density. Therefore, biochemical examination of certain HapR-controlled cellular phenomena (e.g., protease production; suppression of haemolytic activity) of recombinant strains at high cell density will aid in identifying functionally inactive LuxO mutants.

To pursue our working hypothesis, first, we examined the constitutive behaviour of LuxO_PL91_ in strain MM307, a *luxO* mutant of *V*. *cholerae* El Tor strain C6706 **(S1 Table)**, by measuring protease activity at high cell density. We observed MM307 carrying LuxO_PL91_ exhibited a protease negative phenotype **(Fig 1C)**. This indicates that LuxO_PL91_ retains its constitutively active function even in a different *Vibrio cholerae* strains.

**Fig 2:**
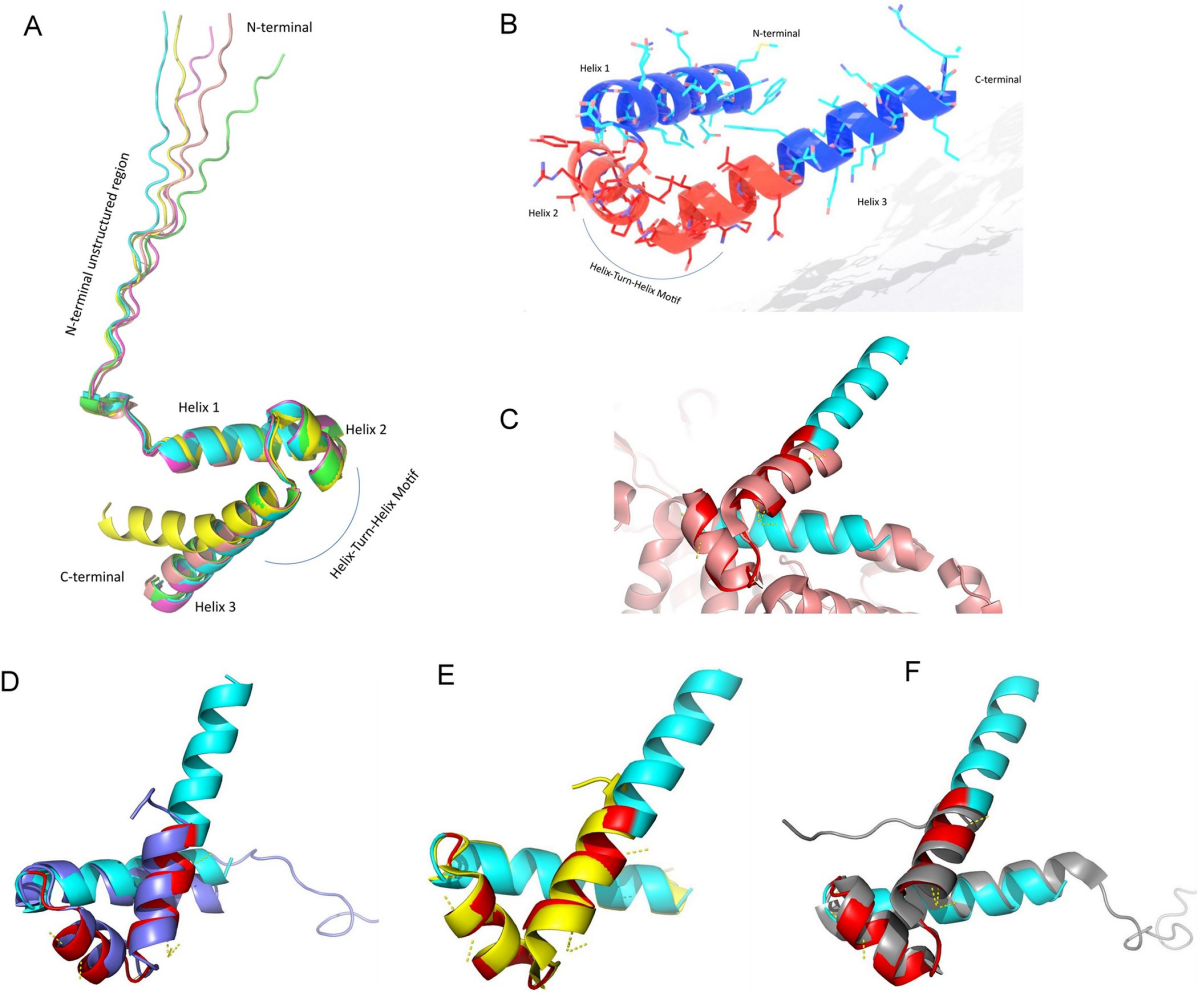
Sequence based modelled 3D structure of LuxO-DBD by ColabFold2 and comparison with its similar structures. (A) Colabfold2 generated 5 model structures of LuxO-DBD (residue 366–443). (B) HTH motif of modelled LuxO-DBD. (Red region selected for Alanine Scanning). (C, D, E, and F) Superimposed LuxO-DBD Model structure with similar structures available in PDB like 5M7O, 1UMQ, 4L5E, and 2M8G respectively.

The DNA binding domain (DBD) of LuxO situated at the C-terminus of the protein shares several conserved residues with members of NtrC family proteins [20]. Besides conserved, there are non-conserved residues as well in the DNA binding domain of LuxO. The DBD, having a characteristic terminal helix–turn–helix (HTH) motif, usually binds to the two high-affinity cooperative binding sites situated ~100 bp upstream of the σ^54^ binding site [39]. σ^54^ regulation by the protein allows a broad dynamic range of transcription and rapid temporal responsiveness to changing conditions. We have created our model of the C-terminal DNA binding HTH motif of LuxO_PL91_ protein by selecting the last 78 residues (residue numbers 366–443). In our AlphaFold2 model (**Fig 2A**), the initial twenty-five residues (366–390) are unstructured due to a low confidence score. However, the rest of the sequence represents three helices connected by two loop regions. This section has been modelled with a high confidence score, as denoted by the pLDDt score in AlphaFold2. C-terminal last helix is the longest one. In one of the models, this helix is shown to have a bend (**Fig 2A**- yellow coloured). Next, we tried to find similar experimental structures deposited in the PDB to consider its authenticity as an enhancer-binding protein. Interestingly, this model of LuxO C-terminal HTH DNA binding domain (**Fig 2B**) shows significant similarities with several NtrC DBD protein structures (**Fig 2C–2F**) deposited in PDB, as data obtained from the DALI server (**Table 1**). Comparing those structures, it is evident that the residue numbers 409–430 tend to fold as a part of the HTH motif. Upstream of the HTH motif the helix 1 made up of 17 residues does not actively participate in DNA-protein interaction but it may contribute to dimer formation during DNA binding.

**Table 1:** Similar structures present in PDB as compared to LuxO DBD.

Protein	PDB ID	Z-score	RMSD (Å)	Seq Identity (%)
**NtrX, an Unusual Member of the NtrC Family of Response Regulators.**	5M7O	6.5	1.3	28
**DNA binding domain of *A*. *aeolicus* NtrC1** **Transcriptional regulator (NtrC family)**	4L5E	7.3	0.8	21
**DNA binding of the effector domain from the global regulator PrrA (RegA) from *R*. *sphaeroides***	1UMQ	5.3	2.1	23
**DNA-binding domains in σ**^**54**^ **transcriptional activators**	2M8G	5.3	1	23
Fis DNA binding Domain bound to 27 bp optimal binding sequence F1 DNA*	3IV5	6.1	1.1	23
*A*. *aeolicus* NtrC4 DNA-binding domain bound with DNA*	4FTH	5.2	1	28
DNA-binding domain of DeoR in complex with the DNA operator*	7BHY	4.7	2.3	22

**Fig 3:**
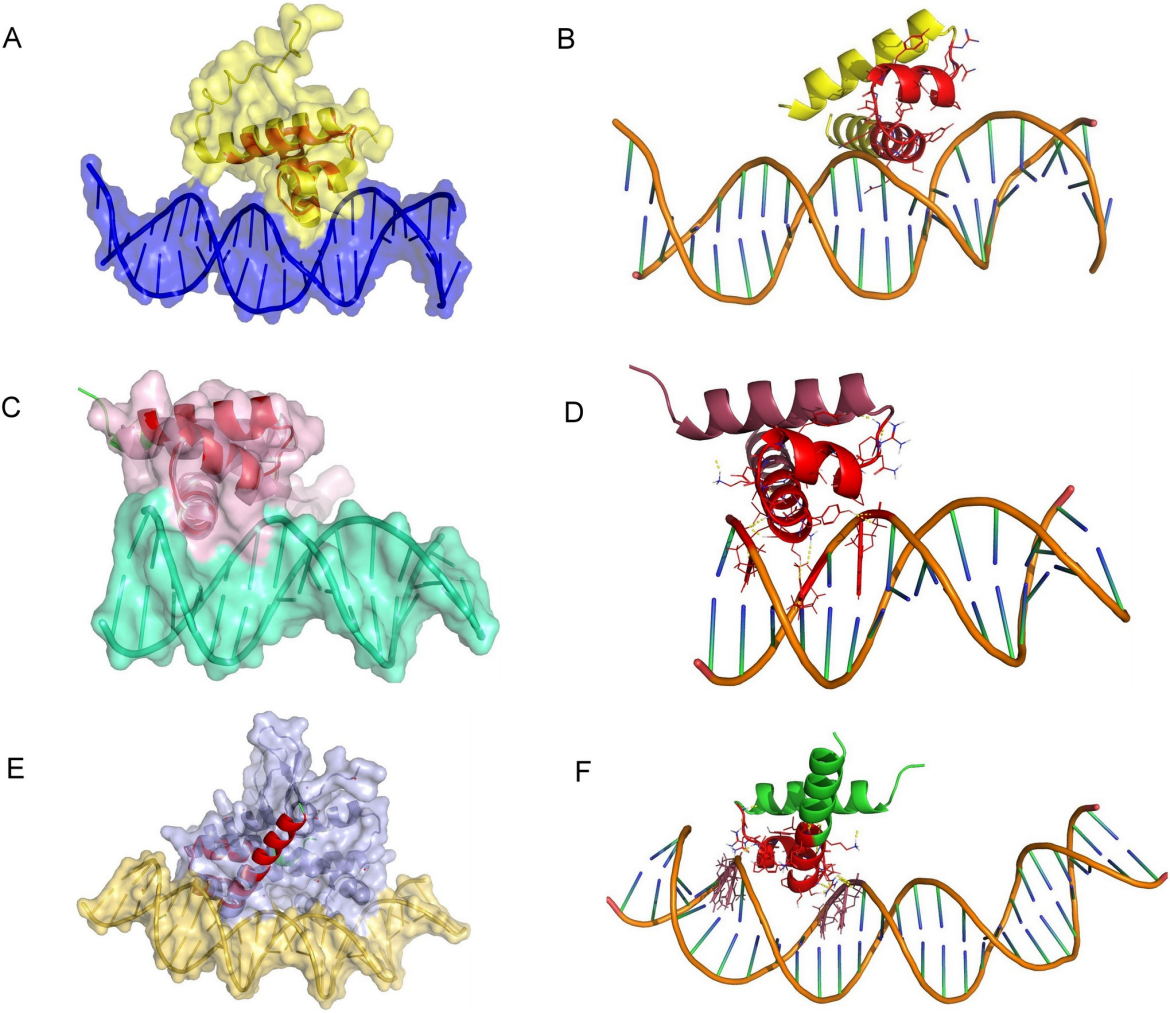
Structural comparison of modelled LuxO DBD with other NtrC like DNA binding protein DNA complex structure. (A, C, and E) LuxO DBD modelled structure (Red) superimposed with DNA binding proteins in the structures (surface representation) 4FTH, 7BHY and 3IV5 respectively. (B, D, and F) Energy minimized LuxO modelled structure with the respective DNA of the PDB structures of 4FTH, 7BHY and 3IV5. (Red region is highlighted that is proximal to DNA and selected for alanine mutations).

According to the previous study of NtrC DBD, a structural homologue of LuxO, the N-terminal helices makes an antiparallel helix bundle with the corresponding helices in the adjacent monomer during DNA binding but do not actively interact with DNA [40]. Despite having the same structural motifs, the C terminal sequence similarities with the iso structure are significantly less (< 30% approx.). Thus, we also tried identifying the structural implications of the amino acids in this region. When the apo structures helped us compare the relevant positions of the amino acids in the HTH motif, we extended our analysis to compare the models with DNA-bound complexes. Three DNA-bound complex structures 4FTH, 7BHY, and 3IV5 were identified from our search results. 4FTH is NtrC4 DBD of *Aquifex aeolicus* bound to double-stranded DNA and 7BHY is *Bacillus subtilis* transcriptional regulator DeoR DBD complexed with DNA, and 3IV5 is an *Escherichia coli* Fis protein DBD bound to F1 DNA. Superimposing the modelled LuxO HTH domain with the NtrC4 DBD (**Fig 3A**), DeoR DBD (**Fig 3C**), and Fis DBD (**Fig 3E**) [21,41] in the above-mentioned complexes, we also identified the residues involved in the DNA-protein interaction. We replaced the modelled LuxO-DBD in the respective DBD proteins and, after energy minimization, analyzed the probable protein and DNA interactions in those complexes (**Fig 3B, 3D and 3F**). In most of the models, residue 420–427 of the third helix shows DNA binding propensities. But the model that we superimposed with the Fis protein (**Fig 3F**), residue 409–413 were also shown to be placed inside the major groove of the DNA. The binding pose of the HTH motif to the DNA groove is also differently observed here. Hence, the region consisting of residue 409 to residue 430 of the HTH motif is present at critical binding positions contributing to DNA interaction. The N-terminal helix 1 does not significantly contribute to DNA binding but may be a part of the C-terminal antiparallel helix bundle formation. Hence, to understand *in vitro*, we selected twenty-two amino acids (residues from 409 to 430) to employ alanine scanning mutagenesis to check the contribution of each amino acid in the LuxO function.

### Alanine scanning mutagenesis to identify critical residues in the DNA binding domain of LuxO: Modulation of quorum sensing mediated phenotypic traits exhibited by recombinant *Vibrio cholerae* strains carrying LuxO alanine mutants

**Fig 4:**
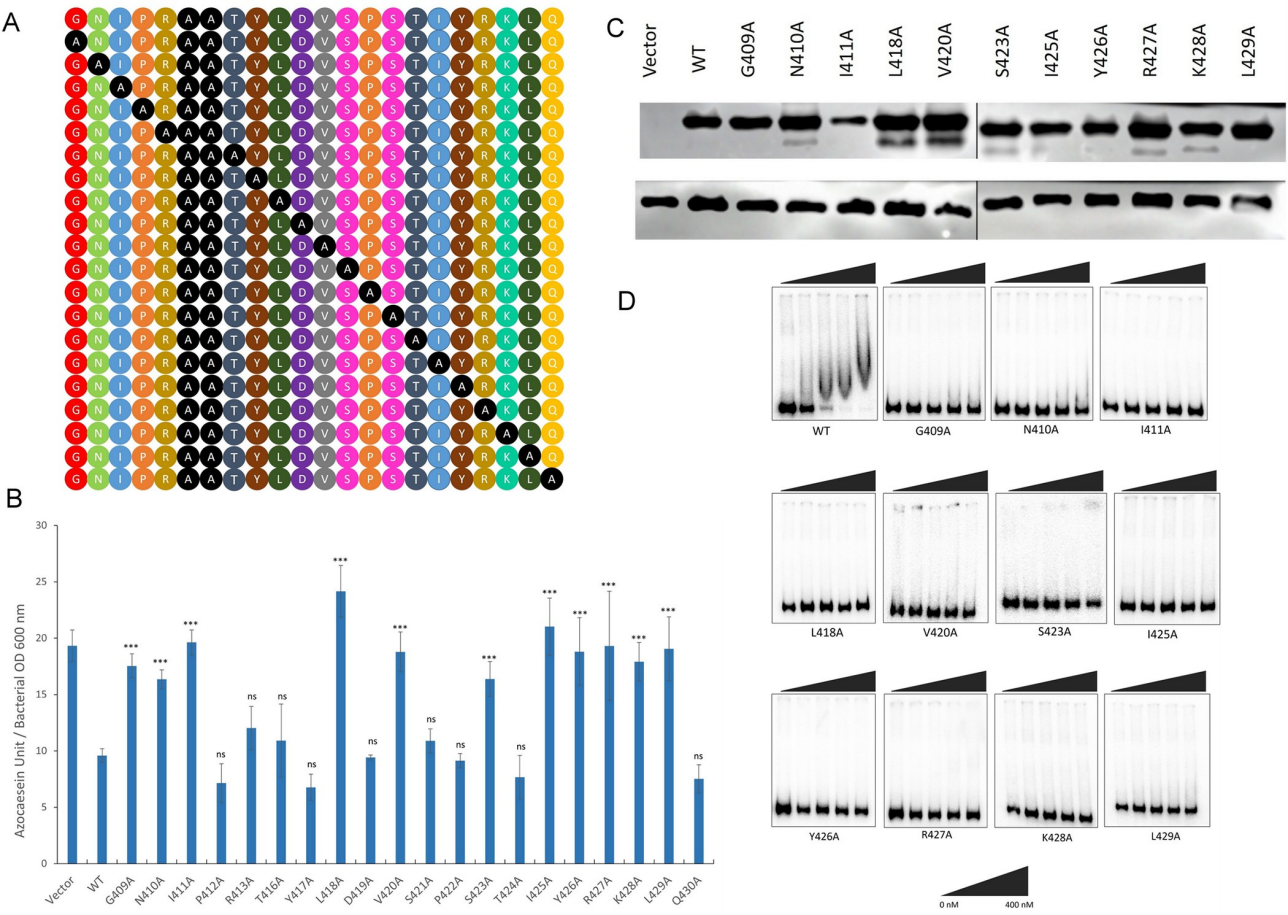
Identification of critical amino acid residues employing alanine scanning mutagenesis. (A) Alanine scanning mutagenesis of the predicted LuxO DNA binding domain (DBD) wherein each residue is mutated to Alanine one at a time. (B) Proteolytic activity demonstrated by the LuxO DBD alanine mutants via azocasein degradation. The enzyme activity is represented as the average of the data values performed (*n = 6*) and is plotted as mean ± SD. Statistical analysis was performed using one-way ANOVA (*—P < 0.05; **—P < 0.01; ***—P < 0.001; ns—not significant). (C) Overnight grown cultures of recombinant MM307 harbouring LuxO_PL91_ FL (WT) and the FLAG-tagged LuxOPL91 DBD alanine mutants with loss in activity, were used to prepare cell lysates, which were separated on 12% SDS-PAGE. The FLAG-tagged LuxO proteins were probed using HRP-conjugated anti-FLAG monoclonal antibody. An anti-RNA polymerase antibody was used for loading control. The upper panel shows the FLAG-tagged LuxO variants, while the lower panel shows RNA polymerase. (D) Electrophoretic mobility shift assay (EMSA) of purified LuxO_PL91_ and its DBD alanine mutants (G409A, N410A, I411A, L418A, V420A, S423A, I425A, Y426A, R427A, K428A and L429A which have lost their constitutive behaviour) were performed with ^32^P labelled promoter region of Qrr4. A solid wedge indicates the concentration of protein used.

The effectiveness of alanine scanning mutagenesis is well established. This simple method facilitates the evaluation of the functional importance of particular amino acids with protein primary sequence [42–44] and also contributes to the generation of high-value novel bio-molecules [45]. Therefore, we next engaged LuxO_PL91_ for systemic alanine scanning mutagenesis and substituted amino acids of the HTH DNA binding domain with alanine residues except two at positions 414 and 415, which already contain alanine residues (**Fig 4A**). To evaluate functionality, each alanine variant was transformed to MM307. As mentioned, protease production is linked to a loss in the constitutive activity of LuxO. All the recombinant strains of MM307 (**S1 Table**) were subjected to protease production at high cell density. We observed protease production in the case of MM307/LuxO^G409A^; MM307/LuxO^N410A^; MM307/LuxO^I411A^; MM307/LuxO^L418A^; MM307/LuxO^V420A^; MM307/LuxO^S423A^; MM307/LuxO^I425A^; MM307/LuxO^Y426A^; MM307/LuxO^R427A^; MM307/LuxO^K428A^; MM307/LuxO^L429A^ strains indicating these eleven LuxO alanine mutants lost their constitutive activity (**Fig 4B**). In other words, these eleven amino acids in the HTH DBD are critical for the DNA binding ability of LuxO_PL91_. Further to evaluate that this loss in constitutive functionality of alanine variants was not linked with protein instability, the above mentioned eleven LuxO mutants were tagged with Flag-epitope as described earlier [25, 43]. Western blot analysis of the flag-tagged protein indicated the stability of these alanine variants, thereby ruling out that loss in activity is not linked to protein stability **(Fig 4C)**. To confirm that flag insertion did not alter the protein functionality, a protease assay was performed. There was no change in protease production by the recombinant strains carrying flag variants of LuxO alanine mutants (**S1 Fig)**.

LuxO binds to the promoter region of Qrr. LuxO, in collaboration with Fis and σ^54^, drives the expression of Qrr sRNAs in a cell density-dependent manner [37,46]. To assess the DNA binding ability, LuxO alanine variants (LuxO_G409A_, LuxO_N410A_, LuxO_I411A_, LuxO_L418A_, LuxO_V420A_, LuxO_S423A_, LuxO_I425A_, LuxO_Y426A_, LuxO_R427A_, LuxO_L428A_ and LuxO_L429A_) that exhibited loss in constitutive activity were purified and subjected to gel shift assay following published protocols [23,37]. All the eleven non-constitutive alanine variants fail to bind the promoter region of Qrr4, further indicating a compromise in DNA binding ability **(Fig 4D).** LuxO acts as an oligomer. There remains a possibility that alanine substitution in the case of inactive variants might affect the oligomeric status, thus affecting the DNA binding ability of these eleven variants. To examine the oligomeric status, all eleven variants were subjected to SDS PAGE under non-reducing conditions. Our gel analysis data indicated no significant difference in oligomeric status among variants and wild-type proteins **(S2A and S2B Fig)**.

**Fig 5:**
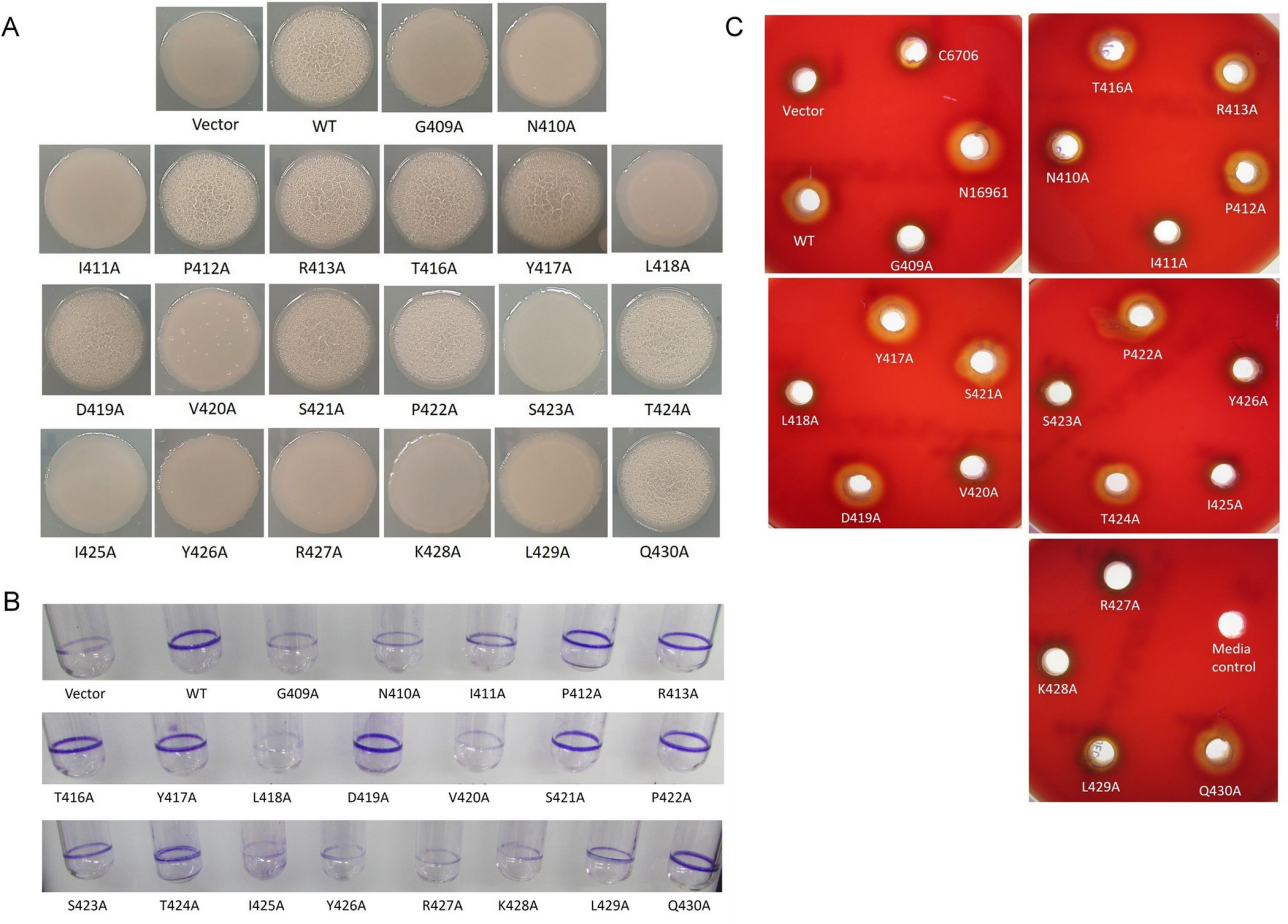
In vivo assays of *V*. *cholerae* recombinant strains to validate the loss of constitutive activity of alanine mutants. Overnight grown cultures of MM307 strain harbouring pKK-3RI (Vector), LuxO _PL91_ (WT), and the LuxO_PL91_ DBD alanine mutants were diluted 1:100 in LB and further grown till the early log phase. (A) Cultures were then spotted over the LB agar plates and then incubated at 37°C for 24 h. Plates were then photographed after the incubation time. (B) For the biofilm growth, cultures were inoculated into 1ml of LB media and grown at 37°C for 18 hr without shaking conditions. After the incubation time, the culture containing planktonic cells was removed, and the biofilm ring was washed twice with 1X.

HapR inhibits biofilm formation [47,48] and haemolysin production at high cell density [49]. Therefore, the absence of HapR and HapR function will lead to biofilm formation and the development of rugose colony morphotype in *Vibrio cholerae* [35,50]. We subjected all recombinant strains of *V*. *cholerae* MM307 carrying LuxO_PL91_ alanine variants for rugose colony and biofilm development. We observed pellicle formation and rugose colonies in protease-negative recombinant strains, while protease-positive strains failed to form pellicles and formed smooth colonies (**Figs 4B, 5A and 5B**). In continuation, we examined the production of haemolysin, another cellular event governed by HapR in these recombinant *V*. *cholerae* strains having LuxO DBD mutants. Other than the primary virulence factor (e.g., cholera toxin), haemolysin also contributes significantly to cholera pathogenesis [51]. The production of haemolysin is repressed by HapR both transcriptionally and post-transcriptionally at high cell density [49]. In other words, haemolysin production at high cell density could also be used as another marker other than suppression of protease production for the constitutive behaviour of LuxO. We adopted an agar plate-based haemolytic assay as published earlier [33] to examine haemolysin production by recombinant *V*. *cholerae* strains carrying alanine variants of LuxO_PL91_. Our result suggested that protease-positive recombinant strains carrying non-constitutive LuxO variant showed less haemolytic activity on the blood agar plate corresponding to protease-negative strains having constitutive LuxO (**Fig 5C**) (**Table 2**).

**Table 2:** Zone of clearance for haemolysis activity shown by LuxO DBD mutants.

Culture	Haemolysis Zone (mm)
**N16961**	3.25 ± 0.25
**C6706**	1 ± 0
**Vector**	0
**WT**	2.5 ± 0.5
**G409A**	0
**N410A**	0.5 ± 0.5
**I411A**	0
**P412A**	2.75 ± 0.25
**R413A**	2.5 ± 0
**T416A**	3 ± 0
**Y417A**	2.75 ± 0.25
**L418A**	0
**D419A**	2.75 ± 0.25
**V420A**	0.5 ± 0.5
**S421A**	2.75 ± 0.25
**P422A**	2.5 ± 0.5
**S423A**	0
**T424A**	2.75 ± 0.25
**I425A**	0.25 ± 0.25
**Y426A**	0.5 ± 0.5
**R427A**	0.25 ± 0.25
**K428A**	0
**L429A**	0.75 ± 0.25
**Q430A**	3 ± 0

PBS solution. The biofilm ring was stained with 0.1% crystal violet for 30 min and then images were taken. (C) From the early log phase 10^7^ bacterial cells were then put into the wells created on the sheep blood agar plates and then incubated at 37°C for the 18 hr. After the incubation period, the zone of haemolytic activity was taken photographically.

**Fig 6:**
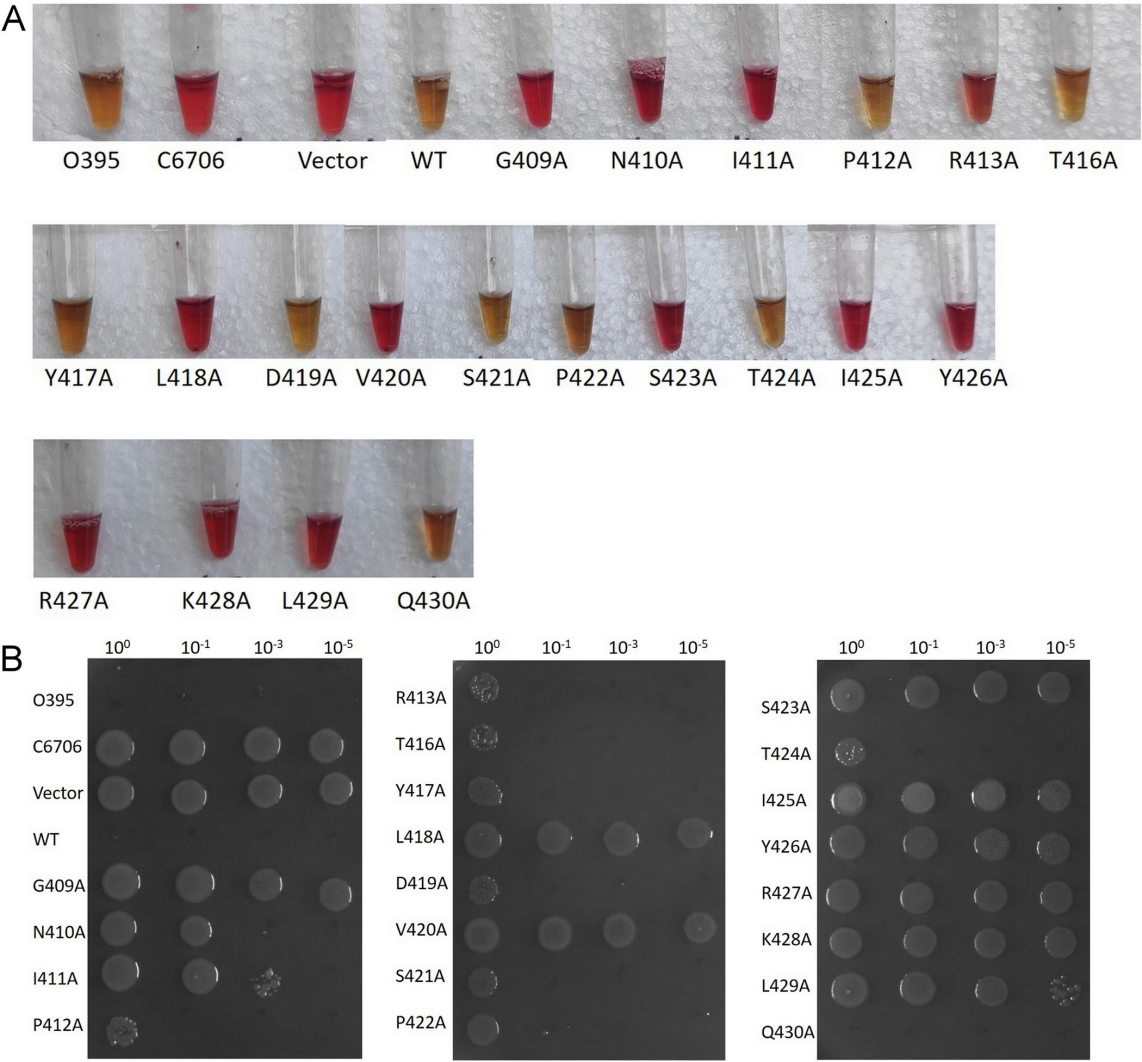
Voges Proskauer test. MM307 carrying pKK-3RI (Vector), LuxO_PL91_ (WT), and LuxO_PL91_ DBD alanine mutants were grown in the presence of LB media containing 1% glucose (LBG). After 12 h of incubation, (A) Voges Proskauer test was performed to examine for acetoin production (B) Spot dilution assay was performed to examine the acid production in culture reflected by cell viability. After being incubated at 37°C for the stipulated time, plates were photographed.

We also examined sugar metabolism of *V*. *cholerae*, which is directly regulated by Qrr sRNA and AphA. It has been previously reported in *V*. *cholerae* that at low cell density, production of organic acids (e.g., formate, acetate and lactate) is higher in cell free supernatants, whereas, at high cell density accumulation of neutral molecules (e.g., acetoin and 2,3-butanediol) takes place [52]. Conversion of pyruvate to 2, 3-butanediol involves enzymes synthesized from gene clusters known as *alsDSO* operon [53–56]. The acetoin operon in *V*. *cholerae* is well characterized and is regulated in a cell density-dependent manner. AphA binds upstream of the acetoin operon and represses the expression of the genes thereby exerting a negative influence on acetoin production [56]. The role of Qrr sRNAs in controlling acetoin biosynthetic gene cluster is also documented where Qrr sRNAs reduces the expression of gene encoding AlsS, leading to the accumulation of organic acids and stress [52]. In other words, constitutively active LuxO will continue to suppress acetoin production through Qrrs. *V*. *cholerae* strains harbouring such a con-LuxO protein will become sensitive to glucose-fermented by-products at high cell density. To examine the link between loss in hyperactivity of LuxO_PL91_ DBD alanine mutants with glucose sensitivity, all recombinant strains were grown in LB containing 1% glucose. We observed acetoin production by the above-mentioned protease-positive recombinant strains carrying non-constitutive LuxO variants (**Fig 6A**). Acetoin data were further corroborated by viability spotting assay where growth was observed with strains with a non-constitutive form of LuxO (**Fig 6B**). Taken together, we identified critical residues in the DNA binding domain of LuxO contributing to its functionality.

## Discussion

The versatile lifestyle of *Vibrio cholerae* strongly depends on the proper functioning of the quorum-sensing signal transduction system. The optimal functioning of this machinery demands an intricate crosstalk among regulatory proteins, small molecules, and small RNAs. One such regulatory protein is LuxO, the low-cell density master regulator. LuxO belongs to the large family of bacterial enhancer-binding proteins. Members of this family are recognized as activators and promote σ^54^ -mediated transcription [6,10]. A detailed structural analysis reveals a three-domain architecture, a domain organization common to all members where the N-terminal acts as a regulatory domain, a central catalytic AAA+ domain, and a C terminal DNA binding domain [10,57]. Based on the biological functions and modes of regulation, members are clustered into five different groups. It should be noted that the DNA binding domain consisting of the helix-turn-helix (HTH) motif is present in all groups except group 5 [10,58,59]. Based on domain organization and modes of regulation, NtrC, the close homolog of LuxO, is categorized into group I of the bEBP family [10]. The ATPase activity of bEBP is crucial for the transition of RNAP- σ^54^ closed complex into the open complex. The conserved GAFTGA signature motif in the AAA+ domain of bEBP members plays an important role in interacting with σ^54^ and this interaction further promotes the isomerization of the closed complex to open complex [11,60–62]. In the case of LuxO_PL91_, the conserved GAFTGA is located at position 195. Since its discovery, many insights into the structural and functional aspects of this protein have been explored, except for the critical residues on the HTH DNA binding domain (DBD). Therefore, we were driven by a desire to identify the important residues in the DBD that contribute to its DNA binding regulatory function. Our interest in LuxO originated when we encountered a natural variant of this protein [18]. In this work, we decided to exploit this hyperactive LuxO (LuxO_PL91_) to understand the role of each amino acid in the DNA binding domain of LuxO. As described previously, the DNA binding domain of LuxO comprises of twenty-two amino acids. Individually mutating these residues to alanine allowed us to study the contribution of each amino acid in the DNA binding ability. All the recombinant *V*. *cholerae* strains carrying these alanine variants were subjected to a series of biochemical and phenotypic assays. LuxO_PL91_ remains constitutively active at high cell density, leading to the constant production of Qrrs and rendering HapR inactive. We found that following eleven alanine mutants (LuxO_G409A_, LuxO_N410A_, LuxO_I411A_, LuxO_L418A_, LuxO_V420A_, LuxO_S423A_, LuxO_I425A_, LuxO_Y426A_, LuxO_R427A_, LuxO_L428A_ and LuxO_L429A_) showed loss of function as they reverted this negative protease production phenotype exhibited by the LuxO_PL91_. It is possible that the loss in function of these eleven alanine variants could be a result of the instability of the proteins under in-vivo conditions. Our FLAG–western data indicated that all recombinant proteins are stable and their intactness is comparable to the control proteins (**Fig 4C**). In other words, loss in function is not linked with the stability of these alanine variants.

Next, our aim was to confirm that this loss in activity of the protein is due to the hindrance in DNA binding faced by the DBD alanine mutants. The EMSA results presented that all these eleven DBD alanine mutants were unable to bind to the designated promoter region, further confirming that these residues might be actively taking part in the DNA binding by LuxO **(Fig 4)**. Further biochemical assays corroborated the protease and EMSA findings **(Figs 5 and 6)**. Our collective data aids in the identification of eleven amino acids that contribute the most to LuxO-DNA binding regulatory function.

Previous works pertinent to LuxO were mainly limited to explaining functional and structural aspects of the R and C domains of the protein. Very little is known about the LuxO DBD domain. Thus, the knowledge of different structural and functional aspects of the DBD of LuxO is mainly corroborated here with another bacterial enhancer binding transcriptional activator NtrC and related proteins. The DBDs of NtrC proteins are close homologs and evolutionary ancestors of the versatile DNA binding-and-bending protein, Fis [63]. In these two types of proteins, a four-helix assembly can be observed at the C-terminal region, contributing to the dimerization of the DBD. This self-dimerization helps in DNA binding by placing other recognition helices of the protein with a spacing close to that of two successive major grooves of the DNA. This is observed in our modelled LuxO structure N terminal helix 1 (**Fig 2B**). This part does not directly bind to DNA but may contribute to the proper placement of the LuxO-DBD. C-terminal HTH motif mainly participates in the DNA-protein association.

NtrC-DNA bound structure and our modelled LuxO was compared to gain an insight about the probable nucleotide binding role of the amino acids present in the HTH motif of LuxO DBD. Association of protein-DNA complex is mainly achieved by the amino acid side chains of the protein and the nearest part of the purine and pyrimidine bases of the DNA. Successful interaction is always dependent on the distance of these two elements. As observed in our 3D model analysis, the C-terminal LuxO region selected for alanine scanning has probable interaction with the DNA groove (highlighted in red in **Fig 2B**). Thus, changing the residues into alanine significantly reduces amino acid side chain and DNA base interaction and restricts the DNA and side chain propensities that, in turn, cause hindrance in the point of contact. In addition to that, intramolecular hydrogen bonding involving the sidechains of a few C-terminal amino acids also contributes to stabilising the HTH motif and maintaining the flexibility of the long helix. All these above-mentioned factors contribute to structural alterations and disruption of LuxO functionalities in the C-terminal mutant variants.

Literature is replete with examples of various types of DNA-binding mutants including partial and complete inactive forms to altered specificity variants [64–66]. Interestingly, in some cases, mutants bind altered DNA sequence better than the wild type DNA sequence [64,65]. In another case, a completely defective DNA binding mutant was exploited to gain insight on its interaction with RNA polymerase [67]. In case of LuxO, we got all completely inactive mutants. It would be interesting to examine if these inactive variants can recognize an altered promoter region of Qrrs. Additional studies are necessary to address these issues.

**Fig 7:**
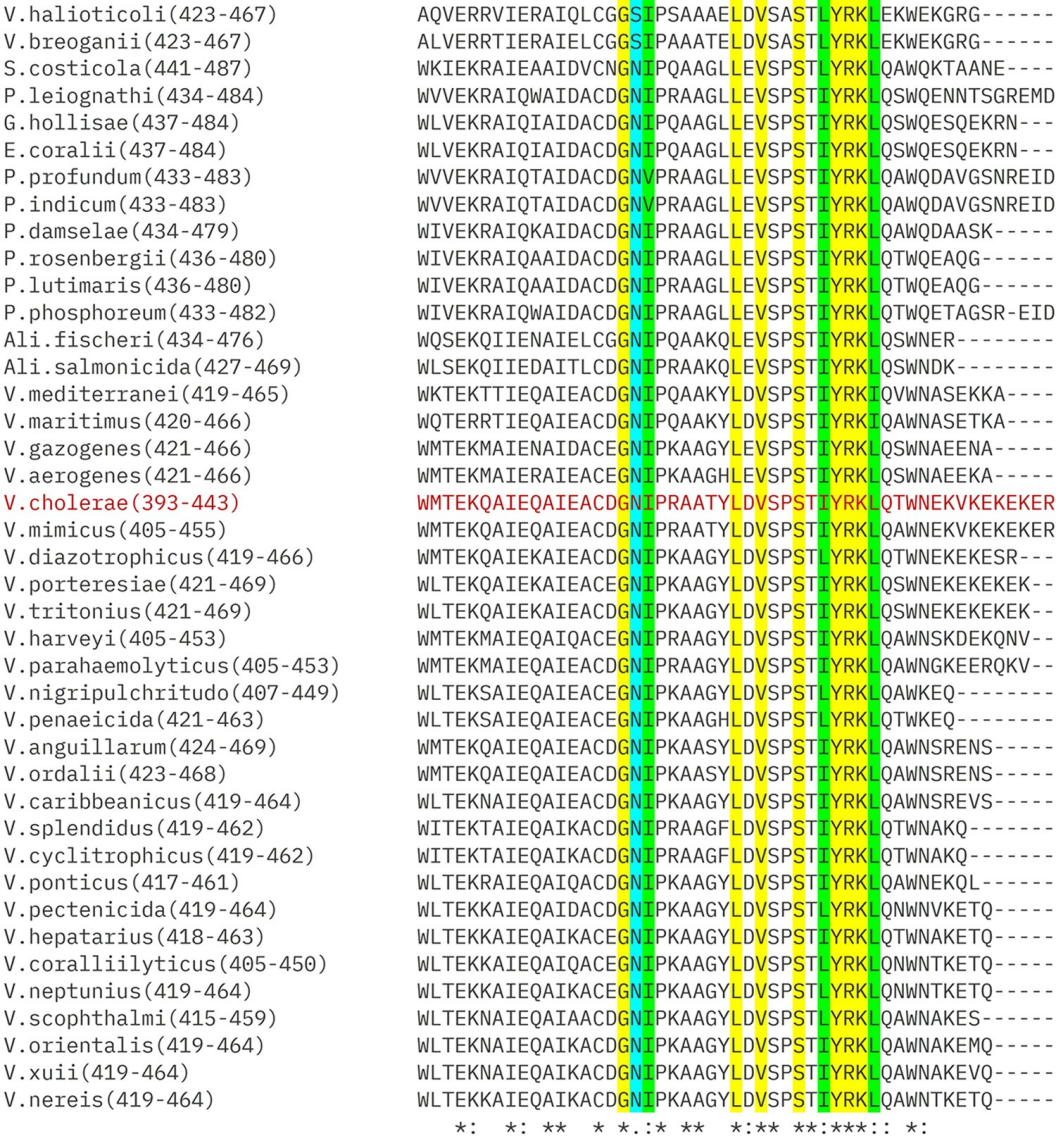
Multiple Sequence Alignment of C terminal region of LuxO across *Vibrionaceae* family. C-terminal region of LuxO proteins from species belonging to different clades of the *Vibrionaceae* family was performed using Clustal Omega. The shaded portions highlight the key residues which are facilitate DNA binding by LuxO (*V*. *cholerae*). The yellow shaded residues are conserved throughout the selected species. The green shaded residues are slightly conserved while the cyan shaded residues are non-conserved.

It has been nearly two decades since the discovery of the helix-turn-helix (HTH), and a lot of data is now available from numerous intense studies on this particular domain [68]. The HTH domain comprises a tri-helical bundle, where the 3^rd^ helix acts as a recognition helix. Out of the eleven critical residues identified in LuxO in this study, six residues (Ser423, Ile425, Tyr426, Arg427, Lys428 and Leu429) belong to the α helix 3 of the HTH motif. It is also registered that certain amino acids (e.g., glycine, arginine, lysine, tyrosine) are contributing the most [69]. Glycine is the most frequently occurring residue at the first position of HTH [68]. As expected, the first residue of the LuxO HTH domain is glycine (G^409^), and substituting this glycine for alanine (G409A) results in a non-functional LuxO variant. ClustalW analysis of LuxO proteins from members of the *Vibrionaceae* family also shows high conservation of glycine at the beginning of HTH (**Fig 7**). Though other residues are conserved, there exists a good number of non-conserved residues in the HTH domain of LuxO from other *Vibrio* species. It would be interesting to examine the role of non-conserved residues in the HTH domain of LuxO protein in the *Vibrionaceae* family. This warrants further investigation.

The accession id are as follows: *Vibrio halioticoli* (WP_023405612.1), *Vibrio breoganii* (WP_102483927.1*)*, *Aliivibrio fischeri* (USR96395.1), *Aliivibrio salmonicida* (CAQ79559.1, *Salinivibrio costicola* (WP_236719540.1), *Grimontia hollisae* (STO57995.1), *Enterovibrio coralii* (WP_067414342.1), *Photobacterium damselae* (WP_272468427.1), *Photobacterium rosenbergii* (WP_317523309.1), *Photobacterium lutimaris* (WP_107348500.1), *Photobacterium phosphoreum* (BAF43691.1), *Photobacterium profundum* (WP_181320793.1), *Photobacterium indicum* (WP_211321716.1), *Photobacterium leiognathi* (WP_318510080.1), *Vibrio mediterranei* (WP_171393353.1), *Vibrio maritimus* (GAL37339.1), *Vibrio nigripulchritudo* (CCO57074.1), *Vibrio penaeicida* (WP_126606785.1), *Vibrio gazogenes* (WP_072962825.1), *Vibrio aerogenes* (WP_073605359.1), *Vibrio harveyi* (AAD12736.1), *Vibrio parahaemolyticus* (AEL22994.1), *Vibrio cholerae* (AAY45912.1), *Vibrio mimicus* (QXC59042.1), *Vibrio diazotrophicus* (RAS67810.1), *Vibrio porteresiae* (WP_261893081.1), *Vibrio tritonius* (WP_068714228.1), *Vibrio splendidus* (UWZ96574.1), *Vibrio cyclitrophicus* (WP_016790353.1), *Vibrio scophthalmi* (WP_334552735.1), *Vibrio anguillarum* (AEH33639.1), *Vibrio ordalii* (WP_286365759.1), *Vibrio caribbeanicus* (WP_039954694.1), *Vibrio orientalis* (WP_004418306.1), *Vibrio xuii* (KOO14497.1), *Vibrio ponticus* (WP_123780404.1), *Vibrio pectenicida* (WP_171359886.1), *Vibrio hepatarius* (WP_167419561.1), *Vibrio nereis* (WP_053396495.1), *Vibrio corallilyticus* (EEX31766.1), *Vibrio neptunius* (QXX07694.1).

## Supporting information

S1 Fig Protease assay of the LuxO DBD loss of function FLAG tagged variants. Protease activity of the overnight-grown LuxO DBD loss of function FLAG variants was simultaneously measured. The enzyme activity is represented as the average of the data values performed (*n = 6*) and is plotted as mean ± SD. Statistical analysis was performed using one-way ANOVA (***—P < 0.001).
(TIF)

S2 Fig SDS Gel images of purified LuxO DBD loss of function mutants. (A) Equal amount (6 μg) of purified LuxO DBD mutants were electrophoresed on 12% SDS-poly acrylamide gel. Samples were prepared without any reducing agent (DTT) to visualize the conformational states of the proteins. (B) SDS PAGE of purified LuxO DBD mutants was performed. Equal amounts of purified proteins were loaded having DTT as reducing agent.
(TIF)

S1 Table List of bacterial strains and plasmids used in this study. (DOCX)

S2 Table List of oligonucleotides used in this study. (DOCX)

S1 File Raw images. (PDF)
