# Supplementary figures and images for: Identification of critical amino acids in the DNA binding domain of LuxO: Lessons from a constitutive active LuxO
Source: PLoS One. 2024 Sep 17;19(9):e0310444. doi: 10.1371/journal.pone.0310444 (PMC11407668; doi:10.1371/journal.pone.0310444)

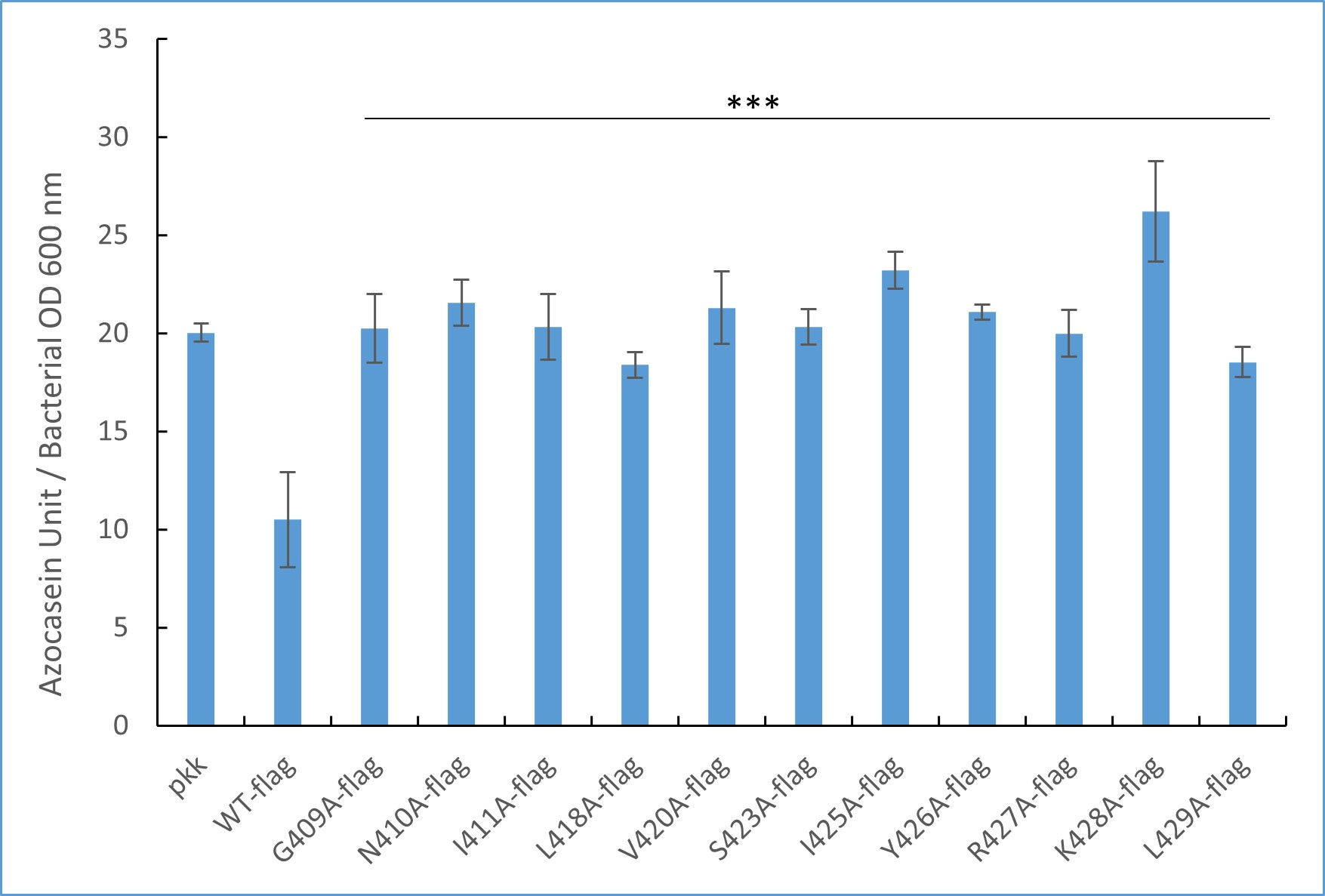

Supplement: S1 Fig — Protease activity of the overnight-grown LuxO DBD loss of function FLAG variants was simultaneously measured. The enzyme activity is represented as the average of the data values performed (n = 6) and is plotted as mean ± SD. Statistical analysis was performed using one-way ANOVA (***—P < 0.001). (TIF) [file pone.0310444.s001.tif]

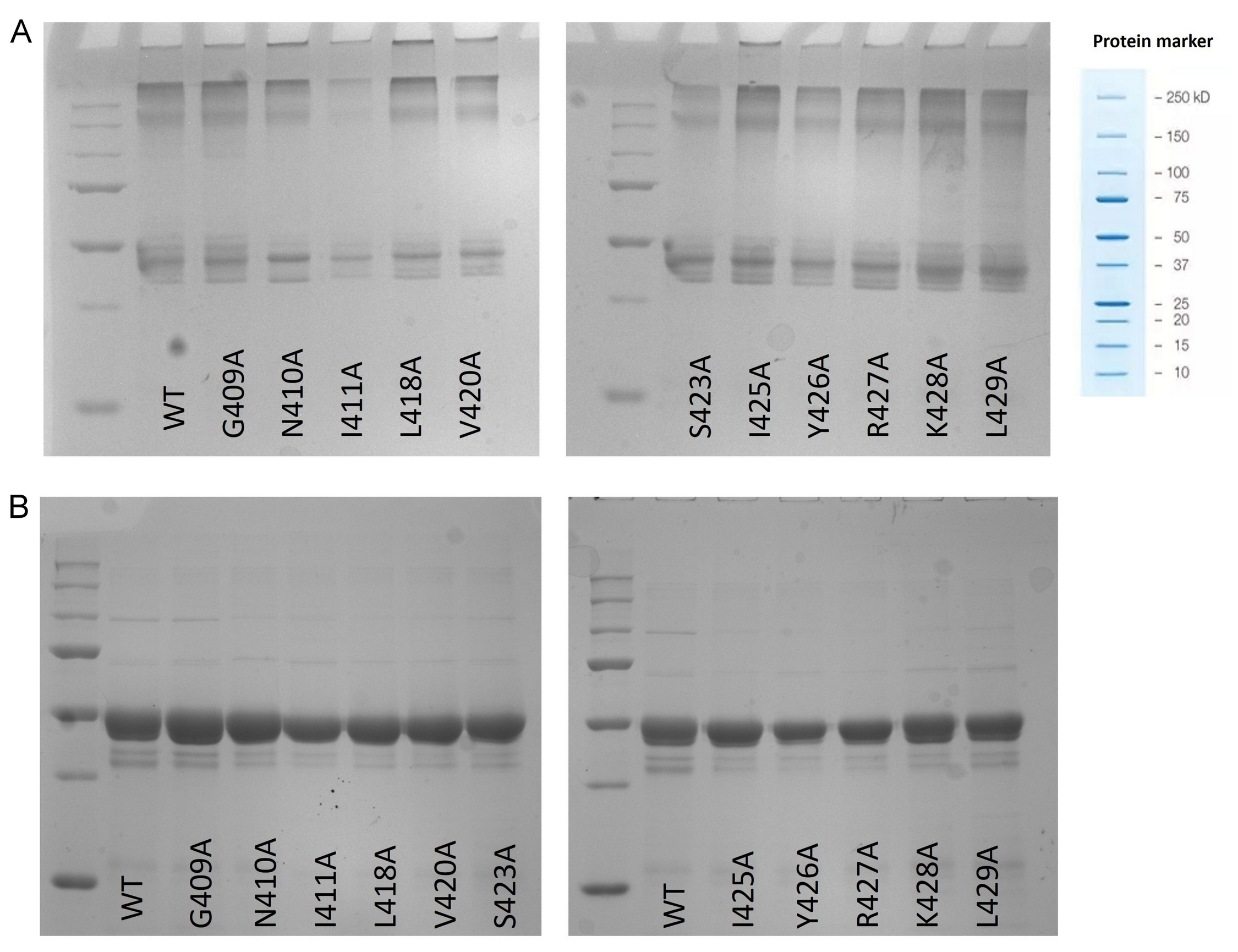

Supplement: S2 Fig — (A) Equal amount (6 μg) of purified LuxO DBD mutants were electrophoresed on 12% SDS-poly acrylamide gel. Samples were prepared without any reducing agent (DTT) to visualize the conformational states of the proteins. (B) SDS PAGE of purified LuxO DBD mutants was performed. Equal amounts of purified proteins were loaded having DTT as reducing agent. (TIF) [file pone.0310444.s002.tif]
