# Supplementary material for: Identification of critical amino acids in the DNA binding domain of LuxO: Lessons from a constitutive active LuxO
Source: PLoS One. 2024 Sep 17;19(9):e0310444. doi: 10.1371/journal.pone.0310444 (PMC11407668; doi:10.1371/journal.pone.0310444)
Supplement: S1 Table — (DOCX) [file pone.0310444.s003.docx]

**S1 Table: List of bacterial strains and plasmids used in this study**

| **Strains** | **Description** | **Source/Reference** |
| --- | --- | --- |
| ***E. coli* strains** |  |  |
| Nova blue | *E. coli* K-12, recA endA, lacIq, lacy, tet^r^ | Novagen |
| BL21(DE3) | *E. coli* B, F– ompT lon, with a λ prophage carrying the T7 RNA polymerase | Novagen |
| ***Vibrio cholerae* strains** |  |  |
| O395 | O1, Classical | Andrew Camilli, Tuft University |
| N16961 | O1 El Tor, Ogawa, Streptomycin resistant (Strep^r^) | Andrew Camilli, Tuft University |
| C6706 | O1 El Tor, variant, Strep^r^ | Ron Taylor, Dartmouth Geisel School of Medicine USA |
| MM307 | O1 El Tor, strain C6706 ΔluxO, Strep^r^ | Dr Jun Zhu, University of Pennsylvania School of Medicine, USA |
| MM307-C | MM307 carrying pKK177-3RI, Ampicillin resistant (Ap ^r^), Streptomycin resistant (Strep^r^) | Dongre et. al, 2011 |
| MM307- pLuxO_PL91_ | MM307 carrying pLuxO_PL91_,  Ap^r^, Strep^r^ | This study |
| MM307- pLuxO_G409A_ | MM307 carrying pLuxO_G409A_,  Ap^r^, Strep^r^ | This study |
| MM307- pLuxO_N410A_ | MM307 carrying pLuxO_N401A_,  Ap^r^, Strep^r^ | This study |
| MM307- pLuxO_I411A_ | MM307 carrying pLuxO_PL91_,  Ap^r^, Strep^r^ | This study |
| MM307- pLuxO_P412A_ | MM307 carrying pLuxO_PL91_,  Ap^r^, Strep^r^ | This study |
| MM307- pLuxO_R413A_ | MM307 carrying pLuxO_PL91_,  Ap^r^, Strep^r^ | This study |
| MM307- pLuxO_T416A_ | MM307 carrying pLuxO_PL91_,  Ap^r^, Strep^r^ | This study |
| MM307- pLuxO_Y417A_ | MM307 carrying pLuxO_PL91_,  Ap^r^, Strep^r^ | This study |
| MM307- pLuxO_L418A_ | MM307 carrying pLuxO_PL91_,  Ap^r^, Strep^r^ | This study |
| MM307- pLuxO_D419A_ | MM307 carrying pLuxO_PL91_,  Ap^r^, Strep^r^ | This study |
| MM307- pLuxO_V420A_ | MM307 carrying pLuxO_PL91_,  Ap^r^, Strep^r^ | This study |
| MM307- pLuxO_S421A_ | MM307 carrying pLuxO_PL91_,  Ap^r^, Strep^r^ | This study |
| MM307- pLuxO_P422A_ | MM307 carrying pLuxO_PL91_,  Ap^r^, Strep^r^ | This study |
| MM307- pLuxO_S423A_ | MM307 carrying pLuxO_PL91_,  Ap^r^, Strep^r^ | This study |
| MM307- pLuxO_T424A_ | MM307 carrying pLuxO_PL91_,  Ap^r^, Strep^r^ | This study |
| MM307- pLuxO_I425A_ | MM307 carrying pLuxO_PL91_,  Ap^r^, Strep^r^ | This study |
| MM307- pLuxO_Y426A_ | MM307 carrying pLuxO_PL91_,  Ap^r^, Strep^r^ | This study |
| MM307- pLuxO_R427A_ | MM307 carrying pLuxO_PL91_,  Ap^r^, Strep^r^ | This study |
| MM307- pLuxO_K428A_ | MM307 carrying pLuxO_PL91_,  Ap^r^, Strep^r^ | This study |
| MM307- pLuxO_L429A_ | MM307 carrying pLuxO_PL91_,  Ap^r^, Strep^r^ | This study |
| MM307- pLuxO_Q430_ | MM307 carrying pLuxO_PL91_,  Ap^r^, Strep^r^ | This study |
| MM307-  LuxO_PL91_ FL | MM307 carrying pLuxO_PL91_ FL, Ap^r^, Strep^r^ | This study |
| MM307-  LuxO_G409A_ FL | MM307 carrying LuxO_G409A_ FL, Ap^r^, Strep^r^ | This study |
| MM307-  LuxO_N410A_ FL | MM307 carrying LuxO_N410A_ FL, Ap^r^, Strep^r^ | This study |
| MM307-  LuxO_I411A_ FL | MM307 carrying LuxO_I411A_ FL, Ap^r^, Strep^r^ | This study |
| MM307-  LuxO_L418A_ FL | MM307 carrying pLuxO_L418A_ FL, Ap^r^, Strep^r^ | This study |
| MM307-  LuxO_V420A_ FL | MM307 carrying LuxO_V420A_ FL, Ap^r^, Strep^r^ | This study |
| MM307-  LuxO_S423A_ FL | MM307 carrying LuxO_S423A_ FL, Ap^r^, Strep^r^ | This study |
| MM307-  LuxO_I425A_ FL | MM307 carrying LuxO_I425A_ FL, Ap^r^, Strep^r^ | This study |
| MM307-  LuxO_Y426A_ FL | MM307 carrying LuxO_Y426A_ FL, Ap^r^, Strep^r^ | This study |
| MM307-  LuxO_R427A_ FL | MM307 carrying LuxO_R427A_ FL, Ap^r^, Strep^r^ | This study |
| MM307-  LuxO_K428A_ FL | MM307 carrying LuxO_K428A_ FL, Ap^r^, Strep^r^ | This study |
| MM307-  LuxO_L429A_ FL | MM307 carrying LuxO_L429A_ FL, Ap^r^, Strep^r^ | This study |

| **Plasmid** | **Description** | **Reference** |
| --- | --- | --- |
| pKK177-3RI | Ap^r^ | Giesla Stroz, National Institute of Health, U.S.A |
| pET28c | N-terminal and C terminal 6XHis tag expression vector, Kanamycin resistant (Kan^r^) | Novagen |
| pLuxO_PL91_ | 1.332 kb LuxO (ORF) of PL91 was amplified and cloned in SmaI-HindIII sites of pKK-3RI | Dongre et al., 2008 |
| pLuxO_G409A_ | pLuxO_PL91_ with Gycine at position 409 mutated to Alanine | This Study |
| pLuxO_N410A_ | pLuxO_PL91_ with Asparagine at position 410 mutated to Alanine | This Study |
| pLuxO_I411A_ | pLuxO_PL91_ with Isoleucine at position 411 mutated to Alanine | This Study |
| pLuxO_P412A_ | pLuxO_PL91_ with Proline at position 412 mutated to Alanine | This Study |
| pLuxO_R413A_ | pLuxO_PL91_ with Arginine at position 413 mutated to Alanine | This Study |
| pLuxO_T416A_ | pLuxO_PL91_ with Threonine at position 416 mutated to Alanine | This Study |
| pLuxO_Y417A_ | pLuxO_PL91_ with Tyrosine at position 417 mutated to Alanine | This Study |
| pLuxO_L418A_ | pLuxO_PL91_ with Leucine at position 418 mutated to Alanine | This Study |
| pLuxO_D419A_ | pLuxO_PL91_ with Aspartate at position 419 mutated to Alanine | This Study |
| pLuxO_V420A_ | pLuxO_PL91_ with Valine at position 420 mutated to Alanine | This Study |
| pLuxO_S421A_ | pLuxO_PL91_ with Serine at position 421 replaced to Alanine | This Study |
| pLuxO_P422A_ | pLuxO_PL91_ with Proline at position 422 mutated to Alanine | This Study |
| pLuxO_S423A_ | pLuxO_PL91_ with Serine at position 423 mutated to Alanine | This Study |
| pLuxO_T424A_ | pLuxO_PL91_ with Threonine at position 424 mutated to Alanine | This Study |
| pLuxO_I425A_ | pLuxO_PL91_ with Isoleucine at position 425 mutated to Alanine | This Study |
| pLuxO_Y426A_ | pLuxO_PL91_ with Tyrosine at position 426 mutated to Alanine | This Study |
| pLuxO_R427A_ | pLuxO_PL91_ with Arginine at position 427 mutated to Alanine | This Study |
| pLuxO_K428A_ | pLuxO_PL91_ with Lysine at position 428 mutated to Alanine | This Study |
| pLuxO_L429A_ | pLuxO_PL91_ with Leucine at position 429 mutated to Alanine | This Study |
| pLuxO_Q430A_ | pLuxO_PL91_ with Glutamine at position 430 mutated to Alanine | This Study |
| pluxO_PL91_ FL | 1.332 kb LuxO (ORF) of PL91, FLAG tagged at N-terminal and cloned in  SmaI-HindIII sites of pKK-3RI | This Study |
| pLuxO_G409A_ FL | pLuxO_G409A_ FLAG tagged at N-terminal | This Study |
| pLuxO_N410A_ FL | pLuxO_N410A_ FLAG tagged at N-terminal | This Study |
| pLuxO_I411A_ FL | pLuxO_I411A_ FLAG tagged at N-terminal | This Study |
| pLuxO_L418A_ FL | pLuxO_L418A_ FLAG tagged at N-terminal | This Study |
| pLuxO_V420A_ FL | pLuxO_V420A_ FLAG tagged at N-terminal | This Study |
| pLuxO_S423A_ FL | pLuxO_S423A_ FLAG tagged at N-terminal | This Study |
| pLuxO_I425A_ FL | pLuxO_I425A_ FLAG tagged at N-terminal | This Study |
| pLuxO_Y426A_ FL | pLuxO_Y426A_ FLAG tagged at N-terminal | This Study |
| pLuxO_R427A_ FL | pLuxO_R427A_ FLAG tagged at N-terminal | This Study |
| pLuxO_K428A_ FL | pLuxO_K428A_ FLAG tagged at N-terminal | This Study |
| pLuxO_L429A_ FL | pLuxO_L429A_ FLAG tagged at N-terminal | This Study |
| LuxO_PL91_ pET28c | 1.332 kb LuxO (ORF) of PL91 was amplified and cloned between NdeI-XhoI sites of pET28c | This Study |
| LuxO_G409A_ pET28c | LuxO_PL91_ carrying G409A mutation cloned in pET28c | This Study |
| LuxO_N410A_ pET28c | LuxO_PL91_ carrying N410A mutation cloned in pET28c | This Study |
| LuxO_I411A_ pET28c | LuxO_PL91_ carrying I411A mutation cloned in pET28c | This Study |
| LuxO_L418A_ pET28c | LuxO_PL91_ carrying L418A mutation cloned in pET28c | This Study |
| LuxO_V420A_ pET28c | LuxO_PL91_ carrying V420A mutation cloned in pET28c | This Study |
| LuxO_S423A_ pET28c | LuxO_PL91_ carrying S423A mutation cloned in pET28c | This Study |
| LuxO_I425A_ pET28c | LuxO_PL91_ carrying I425A mutation cloned in pET28c | This Study |
| LuxO_Y426A_ pET28c | LuxO_PL91_ carrying Y426A mutation cloned in pET28c | This Study |
| LuxO_R427A_ pET28c | LuxO_PL91_ carrying R427A mutation cloned in pET28c | This Study |
| LuxO_K428A_ pET28c | LuxO_PL91_ carrying K428A mutation cloned in pET28c | This Study |
| LuxO_L429A_ pET28c | LuxO_PL91_ carrying L429A mutation cloned in pET28c | This Study |
