# Supplementary material for: Identification of critical amino acids in the DNA binding domain of LuxO: Lessons from a constitutive active LuxO
Source: PLoS One. 2024 Sep 17;19(9):e0310444. doi: 10.1371/journal.pone.0310444 (PMC11407668; doi:10.1371/journal.pone.0310444)
Supplement: S2 Table — (DOCX) [file pone.0310444.s004.docx]

**S2 Table: List of oligonucleotides used in this study**

| **Name of primer** | **Primer Sequence (5’🡪 3’)** |
| --- | --- |
| LuxO SmaI F | TCCCCCGGGATGGTAGAAGACAC |
| LuxO HindIII R | CCCAAGCTTTTACCGTTCCTTCTC |
| LuxO_G409A_ F | CATCGAAGCGTGTGATGCTAACATTCCTCGCGCCGCGAC |
| LuxO_G409A_ R | GTCGCGGCGCGAGGAATGTTAGCATCACACGCTTCGATG |
| LuxO_N410A_ F | GAAGCGTGTGATGGTGCCATTCCTCGCGCCGCGAC |
| LuxO _N410A_ R | GTCGCGGCGCGAGGAATGGCACCATCACACGCTTC |
| LuxO _I411A_ F | GTGTGATGGTAATGCTCCTCGCGCCGCGAC |
| LuxO_I411A_ R | GTCGCGGCGCGAGGAGCATTACCATCACAC |
| LuxO_P412F_ F | GTGTGATGGTAACATTGCTCGCGCCGCGACCTATC |
| LuxO_P412A_ R | GATAGGTCGCGGCGCGAGCAATGTTACCATCACAC |
| LuxO_R413A_ F | GATGGTAACATTCCTGCCGCCGCGACCTATCTG |
| LuxO_R413A_ R | CAGATAGGTCGCGGCGGCAGGAATGTTACCATC |
| LuxO_T416A_ F | CATTCCTCGCGCCGCGGCCTATCTGGATGTCAG |
| LuxO_T416A_ R | CTGACATCCAGATAGGCCGCGGCGCGAGGAATG |
| LuxO_Y417A_ F | CTCGTGCCGCGACCGCTCTGGATGTCAGCCCGTC |
| LuxO_Y417A_ R | GACGGGCTGACATCCAGAGCGGTCGCGGCACGAG |
| LuxO_L418A_ F | CTCGCGCCGCTACCTATGCAGATGTCAGCCCGTC |
| LuxO_L418A_ R | GACGGGCTGACATCTGCATAGGTAGCGGCGCGAG |
| LuxO_D419A_ F | CGCCGCGACCTATCTAGCAGTCAGCCCGTC |
| LuxO_D419A_ R | GACGGGCTGACTGCTAGATAGGTCGCGGCGCGAG |
| LuxO_V420A_ F | GACCTATCTAGATGCAAGCCCGTCAACCATC |
| LuxO_V420A_ R | GATGGTTGACGGGCTTGCATCTAGATAGGTC |
| LuxO_S421A_ F | CTATCTAGATGTCGCACCGTCAACCATC |
| LuxO_S421A_ R | GATGGTTGACGGTGCGACATCTAGATAG |
| LuxO_P422A_ F | CTATCTGGATGTCAGCGCGTCAACCATCTATCG |
| LuxO_P422A_ R | CGATAGATGGTTGACGCGCTGACATCCAGATAG |
| LuxO_S423A_ F | GACGTCAGCCCGGCAACCATCTATCGCAAG |
| LuxO_S423A_ R | CTTGCGATAGATGGTTGCCGGGCTGACGTC |
| LuxO_T424A_ F | GATGTCAGCCCGTCAGCCATCTATCGCAAGC |
| LuxO_T424A_ R | GCTTGCGATAGATGGCTGACGGGCTGACATC |
| LuxO_I425A_ F | GTCAGCCCGTCAACCGCCTATCGCAAGCTGC |
| LuxO_I425A_ R | GCAGCTTGCGATAGGCGGTTGACGGGCTGAC |
| LuxO_Y426A_ F | GTCCGTCAACCATCGCGCGCAAGCTGCAAAC |
| LuxO_Y426A_ R | GTTTGCAGCTTGCGCGCGATGGTTGACGGAC |
| LuxO_R427A_ F | GTCAACCATCTACGCGAAGCTGCAAACTTG |
| LuxO_R427A_ R | CAAGTTTGCAGCTTCGCGTAGATGGTTGAC |
| LuxO_K428A_ F | CAACCATCTATCGCGCGCTGCAAACTTGGAATG |
| LuxO_K428A_ R | CATTCCAAGTTTGCAGCGCGCGATAGATGGTTG |
| LuxO_L429A_ F | CCATCTATCGCAAAGCGCAAACTTGGAATG |
| LuxO_L429A_ R | CATTCCAAGTTTGCGCTTTGCGATAGATGG |
| LuxO_Q430A_ F | CTATCGCAAACTGGCAACTTGGAATGAAAAAG |
| LuxO_Q430A_ R | CTTTTTCATTCCAAGTTGCCAGTTTGCGATAG |
| LuxO OL FLAG F | ATGACGATGACAAGATGGTAGAAGACACGGCGTC |
| LuxO OL FLAG R | GACGCCGTGTCTTCTACCATCTTGTCATCGTCAT |
| LuxO SmaI FLAG F | TCCCCCGGGATGGACTACAAAGAC |
| LuxO NdeI F | GGGAATTACATATGATGGTAGAAGACAC |
| LuxO XhoI R | CCGCTCGAGCCGTTCCTTCTC |
| pQrr4 SphI F | ACATGCATGCACGATTCCCATGGACATAGCG |
| pQrr4 SalI R | ACGCGTCGACTCTAATAAATGCAGATTACG |
