# Supplementary material for: Identification of critical amino acids in the DNA binding domain of LuxO: Lessons from a constitutive active LuxO
Source: PLoS One. 2024 Sep 17;19(9):e0310444. doi: 10.1371/journal.pone.0310444 (PMC11407668; doi:10.1371/journal.pone.0310444)

Fig 4C: Western blots of flag tagged proteins

Upper left panel : labelling done from left

1st lane: Molecular marker (X)

2nd lane: Vector

3rd lane: WT

4th lane: G409A

5th lane: N410A

6th lane: I411A

7th lane: L418A

8th lane: V420A

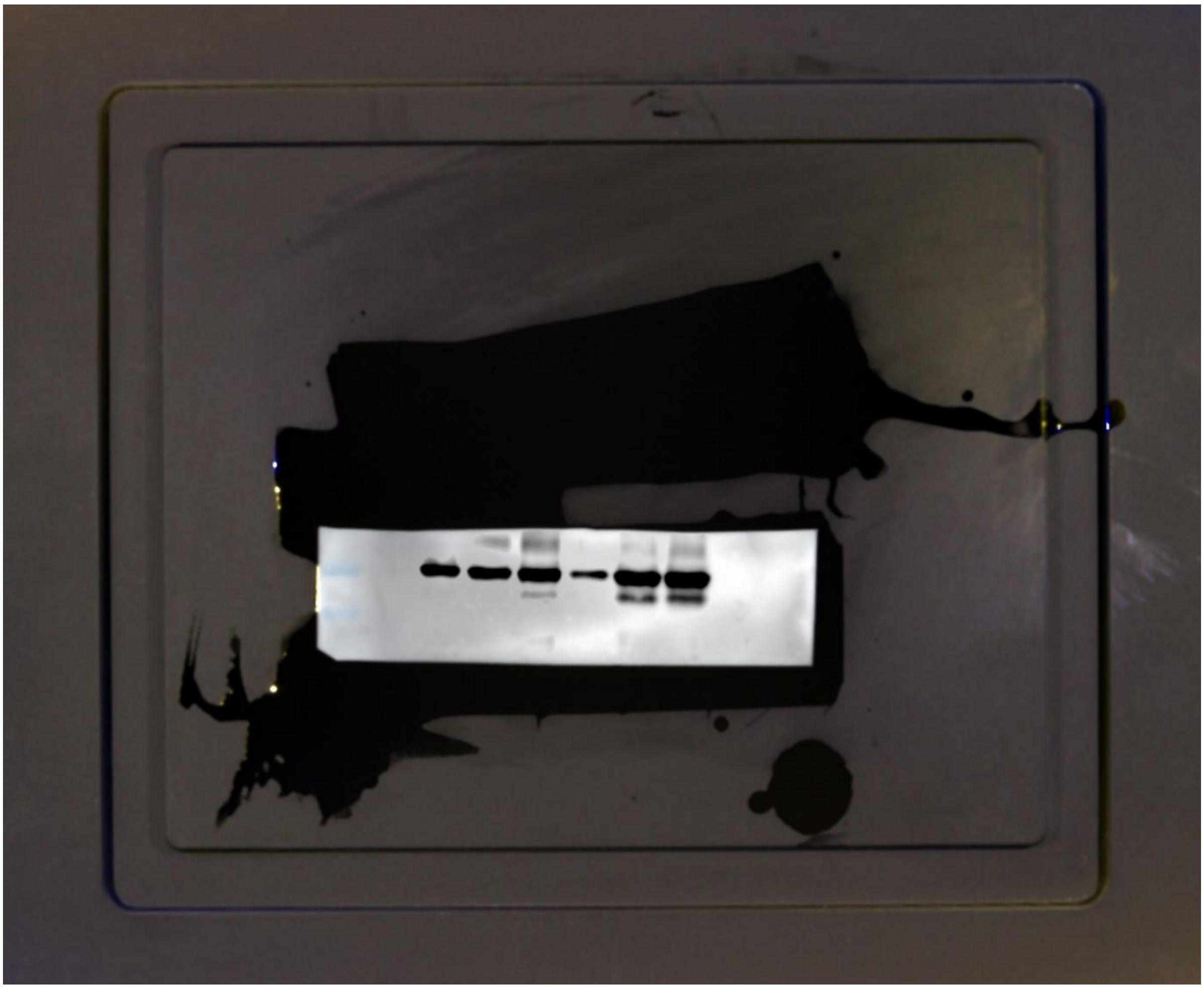

Upper right panel: Lane 4-9 has been considered for this image in the manuscript

1st lane: Molecular marker (X)

2nd lane: Vector (X)

3rd lane: WT (X)

4th lane: S423A

5th lane: I425A

6th lane: Y426A

7th lane: R427A

8th lane: K428A

9th lane: L429A

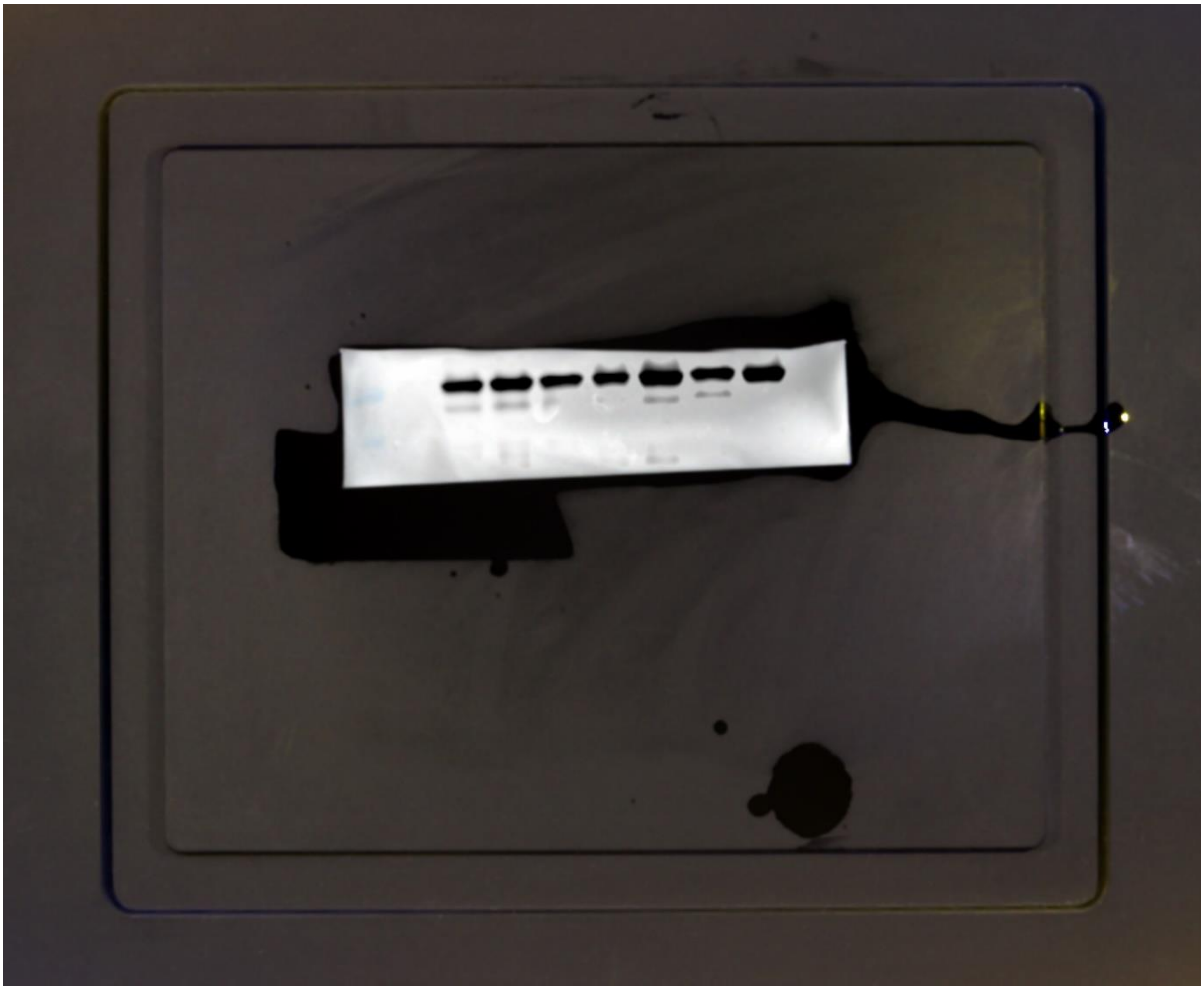

Bottom left panel : labelling done from left

1st lane: Molecular marker (X)

2nd lane: Vector

3rd lane: WT

4th lane: G409A

5th lane: N410A

6th lane: I411A

7th lane: L418A

8th lane: V420A

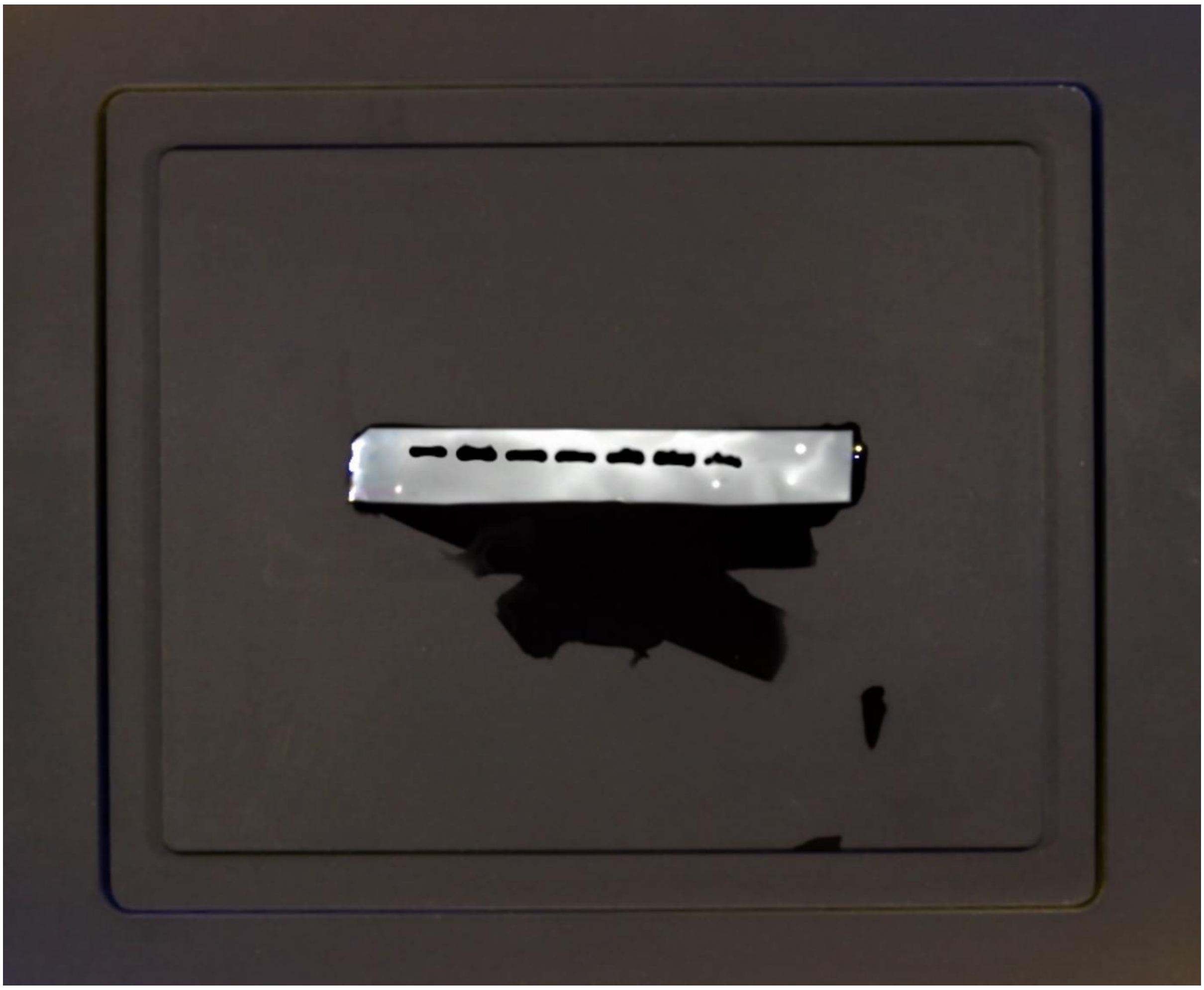

Bottom right panel: Lane 4-9 has been considered for this image in the manuscript

1st lane: Molecular marker (X)

2nd lane: Vector (X)

3rd lane: WT (X)

4th lane: S423A

5th lane: I425A

6th lane: Y426A

7th lane: R427A

8th lane: K428A

9th lane: L429A

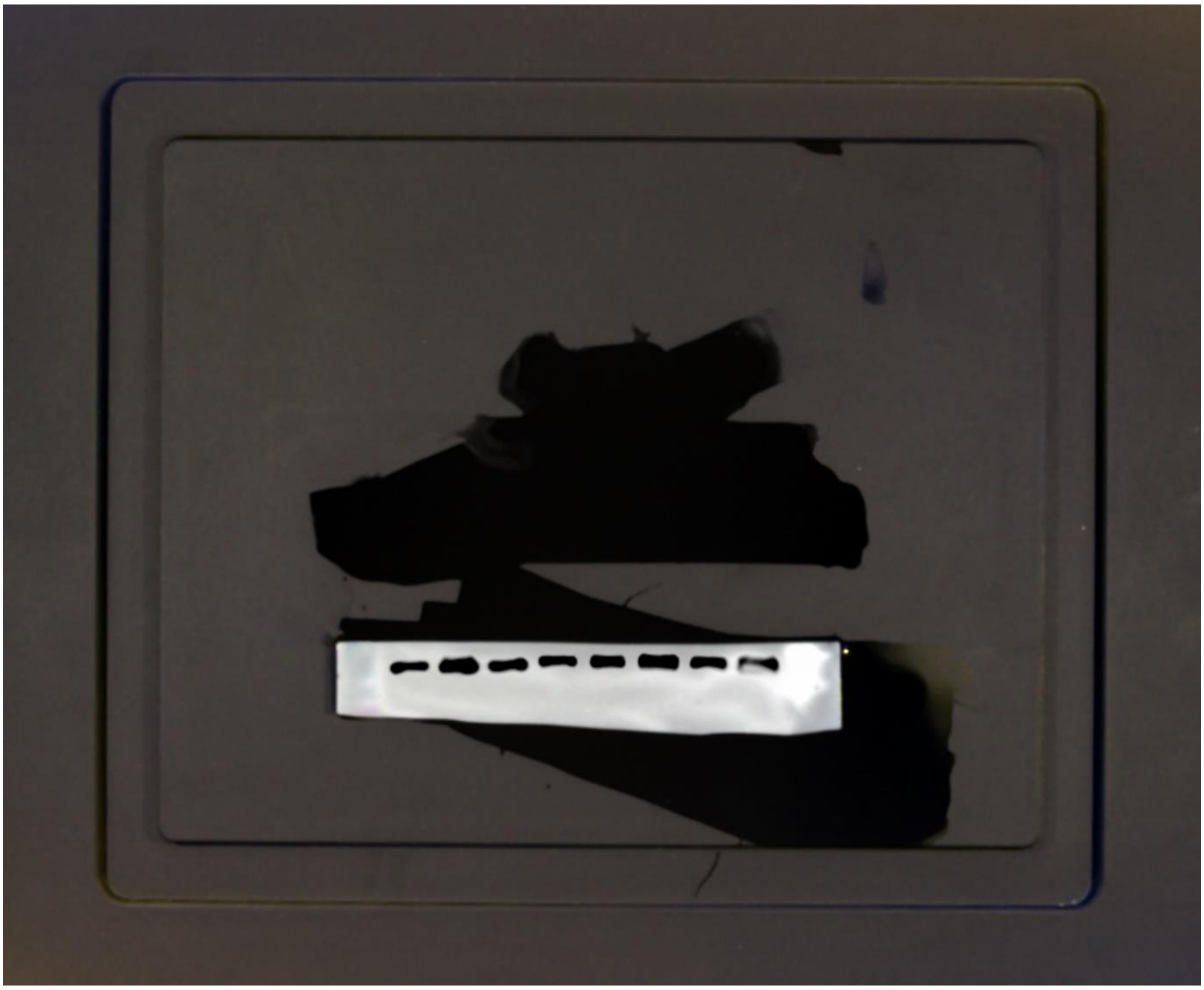

S2 Fig A

Left panel

1st lane: Molecular marker

2nd lane: WT

3rd lane: G409A

4th lane: N410A

5th lane: I411A

6th lane: L418A

7th lane: V420A

8th lane: (X)

9th lane: (X)

10th lane: (X)

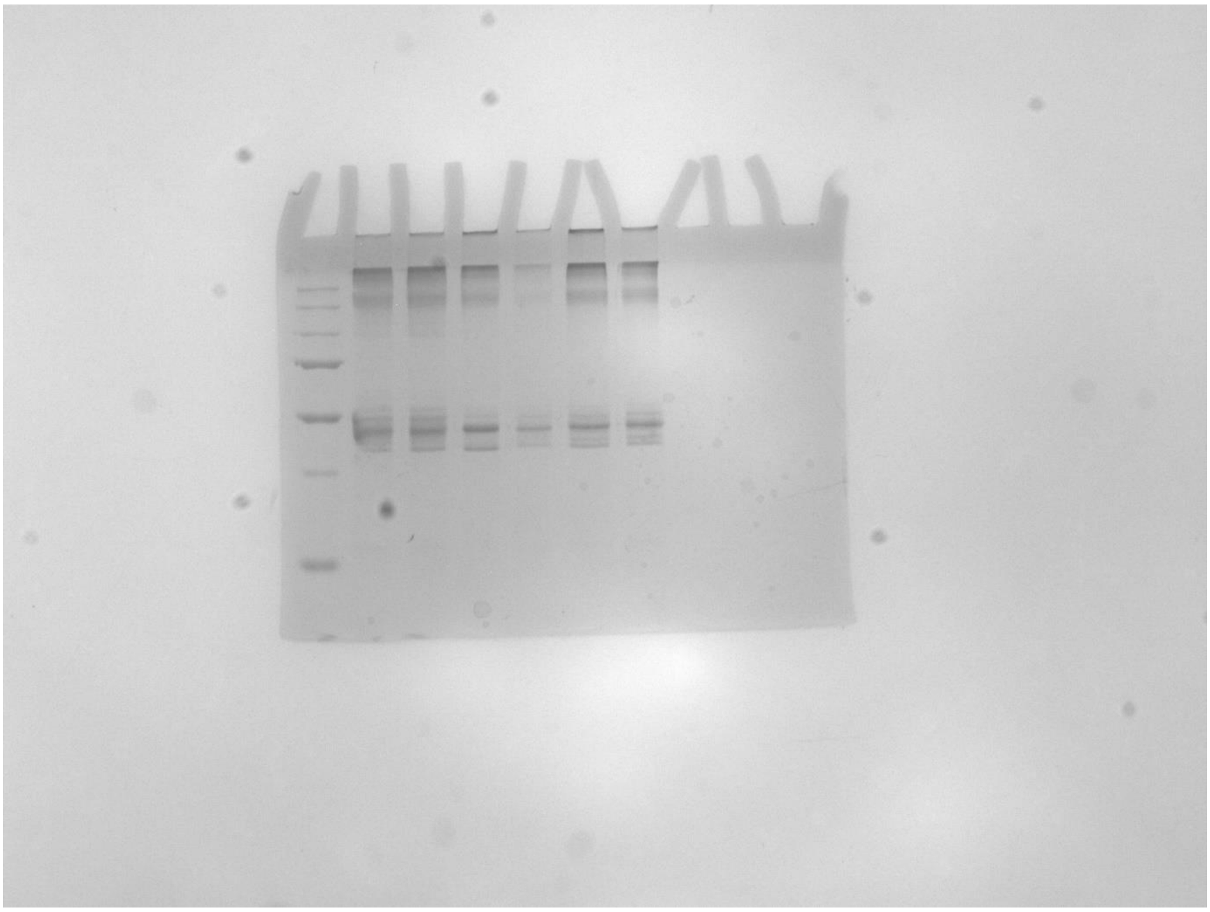

Right panel

1st lane: (X)

2nd lane: Molecular marker

3rd lane: S423A

4th lane: I425A

5th lane: Y426A

6th lane: R427A

7th lane: K428A

8th lane: L429A

9th lane: (X)

10th lane: (X)

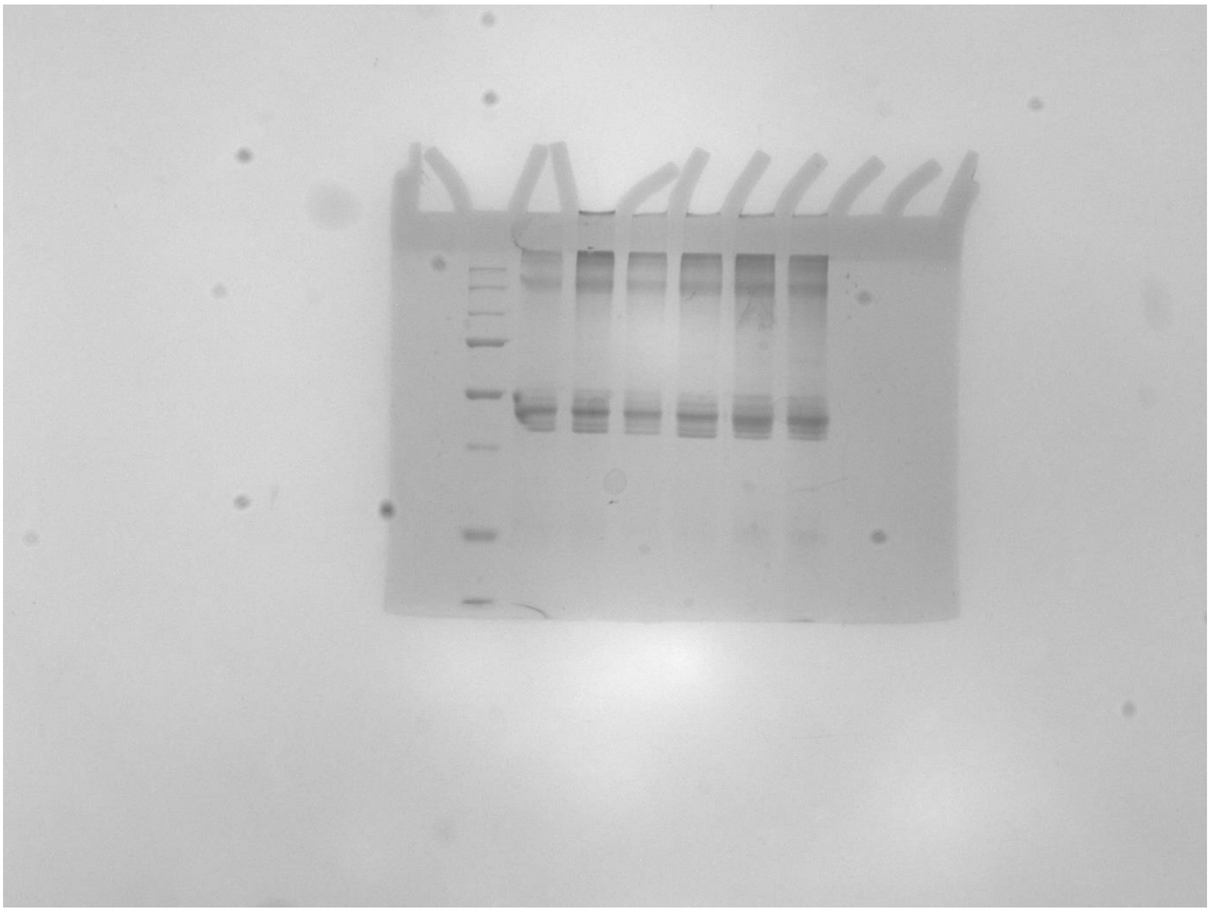

S2 Fig B

Left panel

1st lane: Molecular marker

2nd lane: WT

3rd lane: G409A

4th lane: N410A

5th lane: I411A

6th lane: L418A

7th lane: V420A

8th lane: S423A

9th lane: (X)

10th lane: (X)

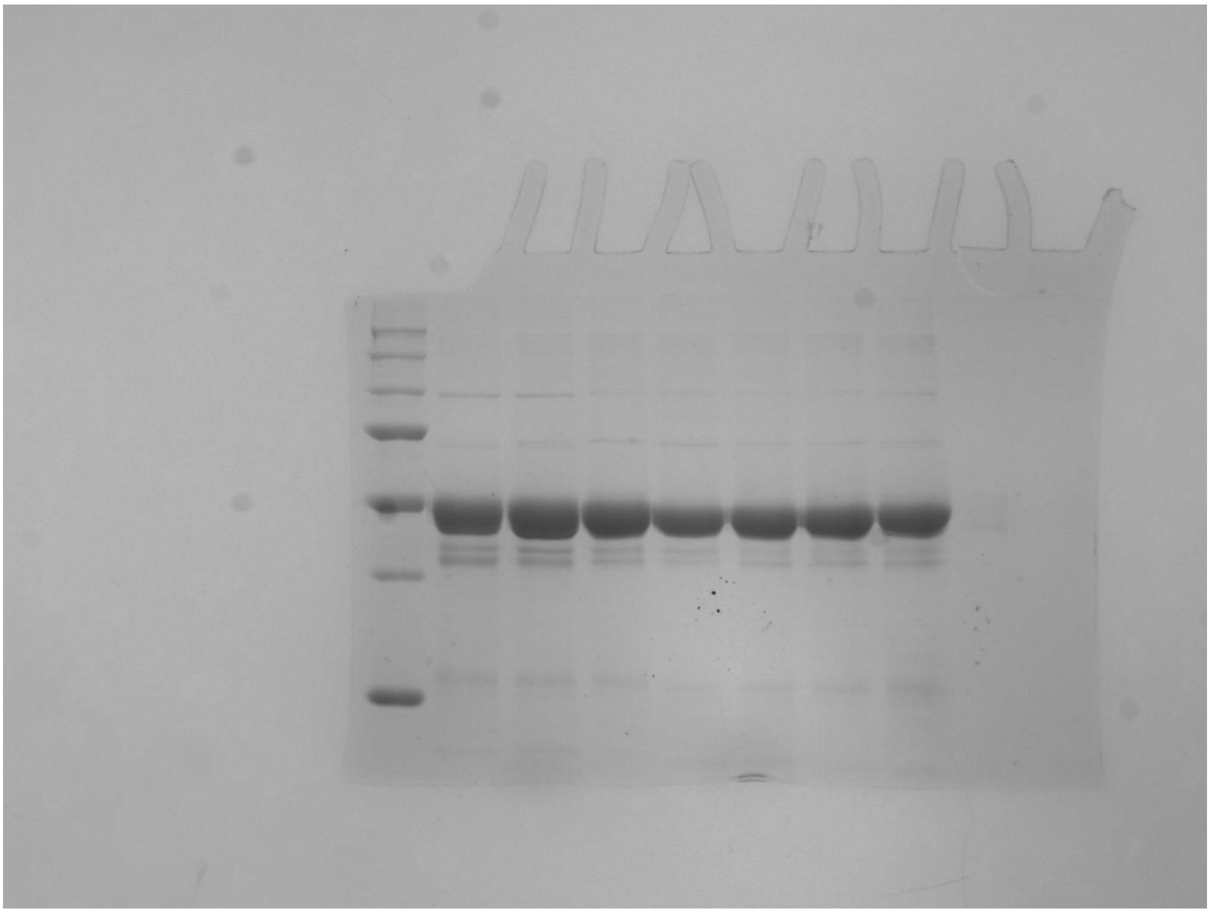

Right panel

1st lane: (X)

2nd lane: Molecular marker

3rd lane: WT

4th lane: I425A

5th lane: Y426A

6th lane: R427A

7th lane: K428A

8th lane: L429A

9th lane: (X)

10th lane: (X)

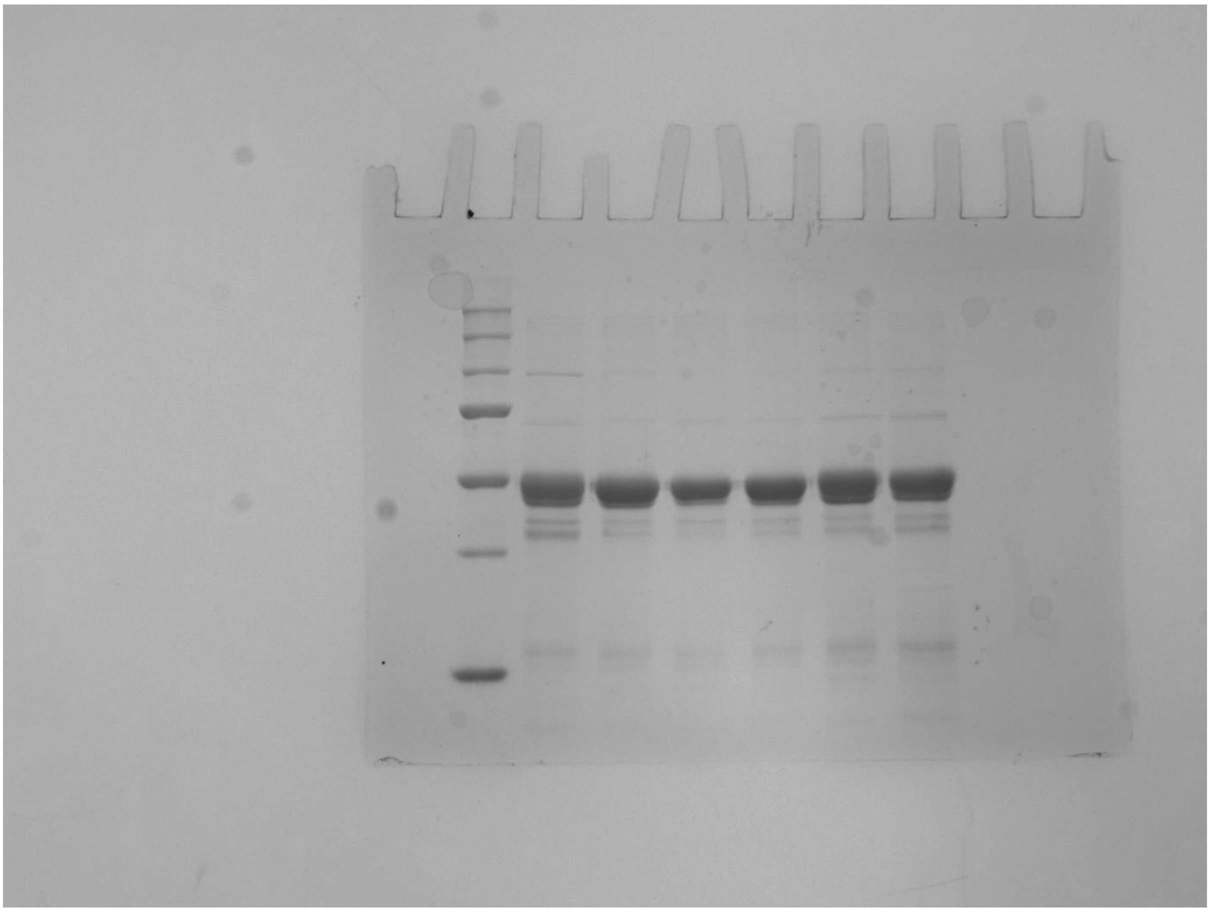

Supplement: S1 File — (PDF) [file pone.0310444.s005.pdf]
